# Reconstruction of the last bacterial common ancestor from 183 pangenomes reveals a versatile ancient core genome

**DOI:** 10.1186/s13059-023-03028-2

**Published:** 2023-08-08

**Authors:** Jason C. Hyun, Bernhard O. Palsson

**Affiliations:** 1 Bioinformatics and Systems Biology Program, University of California, La Jolla, San Diego, CA USA; 2 Department of Bioengineering, University of California, La Jolla, San Diego, CA USA

**Keywords:** Pangenome, Core genome, LBCA, Minimal genome, Metabolism

## Abstract

**Background:**

Cumulative sequencing efforts have yielded enough genomes to construct pangenomes for dozens of bacterial species and elucidate intraspecies gene conservation. Given the diversity of organisms for which this is achievable, similar analyses for ancestral species are feasible through the integration of pangenomics and phylogenetics, promising deeper insights into the nature of ancient life.

**Results:**

We construct pangenomes for 183 bacterial species from 54,085 genomes and identify their core genomes using a novel statistical model to estimate genome-specific error rates and underlying gene frequencies. The core genomes are then integrated into a phylogenetic tree to reconstruct the core genome of the last bacterial common ancestor (LBCA), yielding three main results: First, the gene content of modern and ancestral core genomes are diverse at the level of individual genes but are similarly distributed by functional category and share several poorly characterized genes. Second, the LBCA core genome is distinct from any individual modern core genome but has many fundamental biological systems intact, especially those involving translation machinery and biosynthetic pathways to all major nucleotides and amino acids. Third, despite this metabolic versatility, the LBCA core genome likely requires additional non-core genes for viability, based on comparisons with the minimal organism, JCVI-Syn3A.

**Conclusions:**

These results suggest that many cellular systems commonly conserved in modern bacteria were not just present in ancient bacteria but were nearly immutable with respect to short-term intraspecies variation. Extending this analysis to other domains of life will likely provide similar insights into more distant ancestral species.

**Supplementary Information:**

The online version contains supplementary material available at 10.1186/s13059-023-03028-2.

## Background

Since Darwin’s sketches of diverse species descending from a single ancestor, the growing volume of phenomenological data and, more recently, genomic data, has enabled increasingly comprehensive reconstructions of such “trees of life” [1, 2] and properties of the common ancestor at their roots [3–6]. These modern phylogenetic analyses can incorporate hundreds of thousands of samples to model the evolutionary history of entire domains of life and reconstruct universal common ancestors. However, the increasing scale of data has also produced competing hypotheses regarding universal ancestry such as (1) due to horizontal gene transfer between distant species, evolutionary histories may not be well-represented by a tree [7], and (2) that the last universal common ancestor (LUCA) was not a single organism, but rather a community of interdependent primitive cells [8–10]. These concerns highlight limitations in comprehensively modeling evolutionary history as a tree with individual organisms at each node.

A pangenomic approach offers a more flexible interpretation of the universal common ancestor compatible with these competing hypotheses. The term “pangenome” describes the set of all genes present in a collection of genomes, which includes the core genome (genes present in all genomes) and accessory genes (partially conserved genes comprising the gene-level variability across the genomes). While pangenomes are typically defined from genomes of a single species, the perspective is applicable to any collection of related genomes. The LUCA core genome would represent the set of genes present in all individual LUCA organisms, whether they be members of a single ancestral species or a community of primitive cells. Furthermore, as mobile genetic elements are typically found outside the core genome of a species [11], reconstructing the evolutionary history of core genomes would likely be less confounded by horizontal gene transfer events than using genomes of individual organisms, without requiring predefined marker genes that can limit and bias such analyses [2].

To this end, we apply the following methods to 54,085 genomes spanning 183 species to reconstruct the last bacterial common ancestor (LBCA) core genome. (1) Pangenome construction for each species through protein sequence clustering as previously described [12], (2) a novel core gene identification algorithm based on estimating gene frequencies and genome assembly error rates that maximize the likelihood of observed pangenomes, and (3) reconstruction of ancestral core genomes from the 183 modern core genomes by applying asymmetric Wagner parsimony [13] (minimizing the number of gene gain/loss events between species) to the Web of Life phylogeny [2]. We find that the LBCA core genome, while distinct from any one modern core genome, contains many complete or nearly complete biological systems, especially those related to ribosomal proteins, translation machinery, and metabolic pathways of central carbon metabolism or de novo biosynthetic pathways for nucleotides and amino acids.

## Results

### Construction of 183 pangenomes across the Web of Life phylogenetic tree

**Fig. 1:**
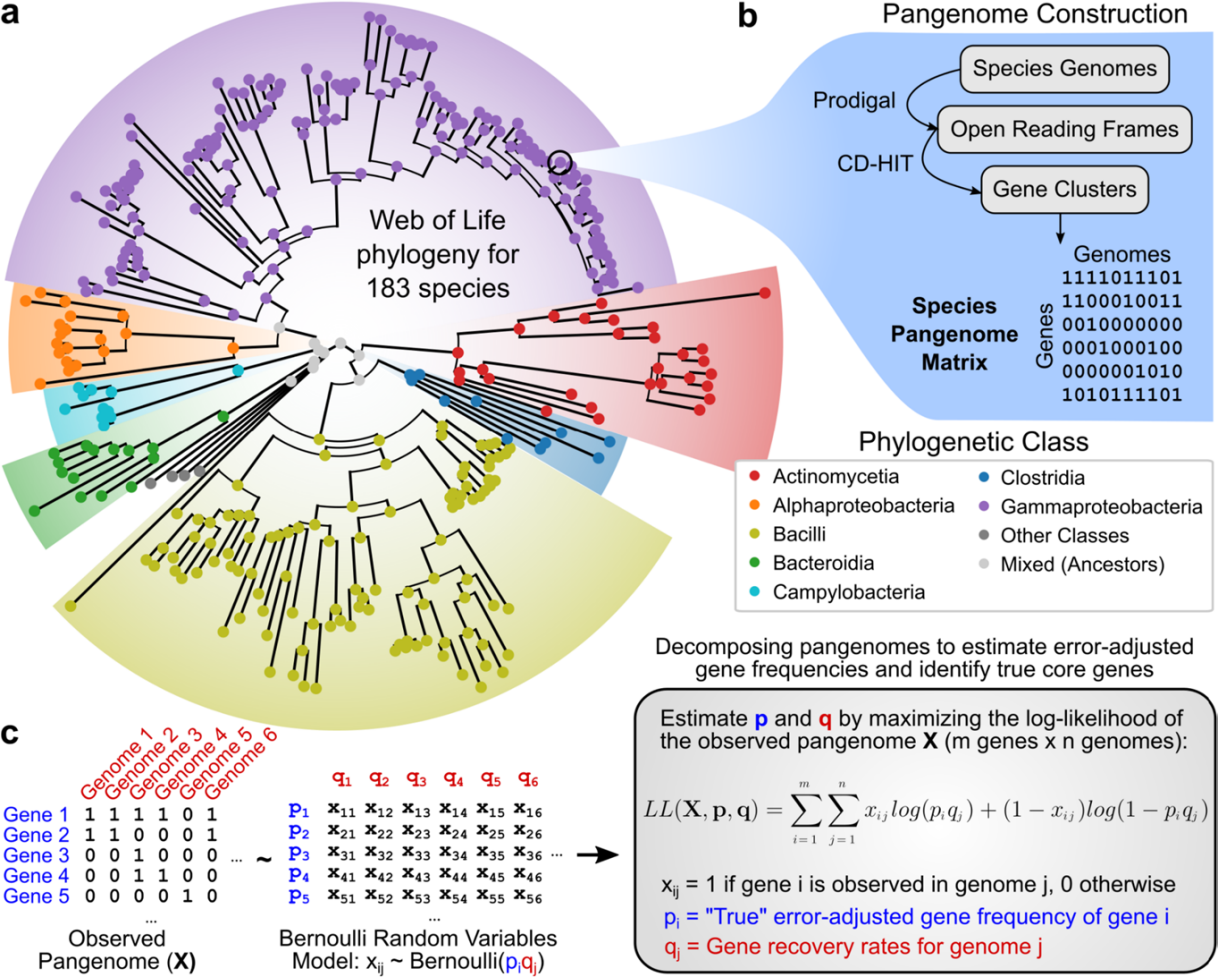
Phylogenetic distribution of 54,085 genomes across 183 species selected for core genome analysis. **a** Phylogenetic tree of species’ representatives with respect to class, with branch lengths derived from the Web of Life tree. Representatives were based on the most recent common ancestors of selected species’ genomes in the original tree. **b** Pangenome construction workflow applied to each species to encode species-wide gene-level variation as binary matrices. **c** Statistical model for estimating each genome’s gene recovery rate (fraction of genes recovered in the final genome assembly) and each gene’s true frequency from species’ pangenome matrices

Starting from Web of Life (WOL) dataset [2], genomes were filtered based on assembly quality, accounting for number of contigs, total length, CheckM contamination, and CheckM completeness [14] (see the “Methods” section for details). Filtering yielded 183 bacterial species as defined by GTDB [15] with at least 50 high-quality genomes each, totaling 54,085 genome assemblies (Additional file 1: Fig. S1, Additional file 2). To integrate species-level properties with the WOL phylogenetic tree which has genomes as leaves, a subtree with species as leaves was extracted as follows. For each species, the most recent common ancestor (MRCA) node of all of its genomes was identified, and the edge of the MRCA was extended by the median distance from the MRCA to the species’ genomes to model a modern representative of that species (Fig. 1a). The final tree with the 183 species as leaves is available in Additional file 3, with species-node mappings in Additional file 2.

Next, open reading frames were annotated in all genomes using Prodigal v2.6.3 [16], and pangenomes were constructed for each species as previously described [12] based on clustering protein sequences with CD-HIT v4.6 [17]. This yielded for each species a sparse binary matrix of presence/absence calls between each genome and gene which could be associated with the species’ corresponding node in the phylogenetic tree (Fig. 1b). These species represent broad ranges in several genetic properties, computed from genome collections ranging from 50 to 4930 genomes per species. Species-wide averages in GC content ranged from 28.0 to 73.4%, genome lengths from 0.82 to 8.39 Mb, and number of genes per genome from 902 to 7515 (Additional file 1: Fig. S1a). Strong correlation was observed between genome length and number of genes (Pearson *r* = 0.991) with a cross-species average and standard deviation of 1080 ± 68 bp per gene, and moderate correlation between either property and GC content (*r* = 0.576 and 0.544, respectively) (Additional file 1: Fig. S1b). Overall, the selected genomes and species capture a substantial degree of genetic diversity in the bacterial domain.

### A model for estimating error-adjusted gene frequencies and identifying core genes

In conducting pangenome analyses, a species’ core genome is defined as the set of genes observed in all its strains. However, due to artifacts in sequencing, assembly, annotation, or clustering, some genes are invariably lost during pangenome construction and a direct application of this definition is too strict to be meaningful for large genome collections (Additional file 1: Fig. S2a). Consequently, many pangenome studies relax this requirement by allowing core genes to be missing from some fraction of the total number of genomes, such as up to 1% by default in the Roary pipeline [18], or up to 5% for “soft-core” genes in the GET_HOMOLOGUES pipeline [19]. However, this proportional cutoff approach assumes that the rate at which genes are lost per genome is the same for all genomes, regardless of the experimental or analytical techniques used.

To address this issue, we developed a model for estimating genome-specific gene recovery rates (fraction of genes not lost to errors and recovered by the final genome assembly) and “true” error-adjusted gene frequencies (fraction of genomes carrying the gene) from a gene by genome presence/absence matrix. Denoting the true frequency of gene *i* as *p*_*i*_ and the gene recovery rate of genome *j* as *q*_*j*_, we model the presence/absence of gene *i* in genome* j* as a Bernoulli random variable with probability *p*_*i*_*q*_*j*_ for all genes and genomes, assuming for simplicity that the two rates are independent. The *p*_*i*_ and *q*_*j*_ are then estimated simultaneously by maximizing the log-likelihood of the observed presence/absence matrix (Fig. 1c).

Applied to the 183 species’ pangenomes, we find that this approach yields robust estimates consistent with existing methods for assessing genome quality and current understanding of core genomes: (1) genome-specific gene recovery rates were correlated with common assembly quality metrics, especially CheckM completeness (Additional file 1: Fig. S2b-d), (2) estimated frequencies were robust to sampling and consistent with the existence of true core genomes (Additional file 1: Fig. S2e, Fig. S3a-b), (3) estimated frequency distributions had a more consistent functional form than raw observed distributions (Additional file 1: Fig. S3c-e), and (4) core genomes identified using estimated frequencies were of similar size to those defined using traditional approaches but with meaningfully different gene content (Additional file 1: Fig. S4). Details are available in Additional file 1: Supplemental Analysis and Additional file 4, and the estimated frequencies for each gene and species are available in Additional file 5.

### Core genome content is relatively stable across species at the level of functional categories but not individual orthogroups

**Fig. 2:**
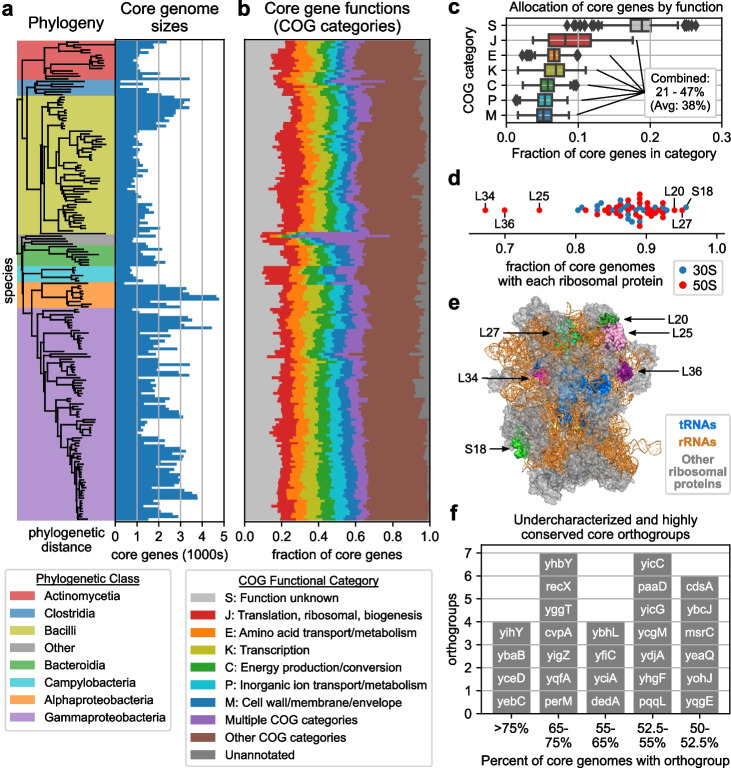
Distribution of gene functions and orthogroups across the core genomes of 183 species. **a** Core genome sizes of each species compared to the phylogenetic tree. **b** Distribution of each species’ core genes to COG functional categories. COG categories with average frequency > 5% are shown, with all others grouped together. **c** Distribution of each species’ core genes to the most commonly observed functional categories. **d** Fraction of core genomes with each bacterial ribosomal protein. **e** Relative positions of the ribosomal proteins appearing in the largest/smallest number of core genomes (structure adapted from PDB:7K00). **f** Under-characterized orthogroups observed in at least 50% of all core genomes

Taking the genes with estimated frequency > 99.99% as our definition of each species’ core genome, functional annotations and orthogroup (OG) assignments were generated for all species’ core genes by applying eggNOG-emapper [20] to each gene’s most common sequence variant. We find that while core genome size varies significantly across species (214 to 4766 genes) and relatively independently of phylogenetic placement (Fig. 2a), the allocation of these genes to different COG functional categories is stable (Fig. 2b, c); principal component analysis (PCA) of these core gene functional category distributions was unable to distinguish different phylogenetic classes (Additional file 1: Fig. S5). Seven COG categories comprised at least 5% of all core genomes on average: S (18.4%; function unknown), J (8.9%; translation, ribosomal structure, and biogenesis), E (6.6%; amino acid transport and metabolism), K (6.6%; transcription), C (5.7%; energy production and conversion), P (5.4%; inorganic ion transport and metabolism), and M (5.3%; cell wall/membrane/envelope biogenesis).

However, while the distribution of core genes to functional categories was stable across species, no individual OGs were found universally in all core genomes. The most strongly conserved OG corresponding to *rpsR* (ribosomal protein S18) was found in 175/183 core genomes, just 28 OGs were found in > 90% of core genomes, and 156 OGs were found in > 80% of core genomes (Additional file 1: Fig. S6a). These strongly conserved core OGs were dominated by ribosomal proteins and other translation-associated genes, with just six in > 90% of core genomes not translation-associated: *ruvA* (resolvasome RuvABC subunit), *nusB* (transcription antitermination protein), *gmk* (guanylate kinase), *atpE* (F_0_F_1_-type ATP synthase subunit K), *atpH* (F_0_F_1_-type ATP synthase delta subunit), and *yajC* (protein translocase subunit). Additionally, this lack of universal core OGs was not sensitive to the 99.99% frequency threshold. No OGs were found in all core genomes even at a 99% threshold, and absences of highly conserved OGs were distributed across a wide range of species (Additional file 1: Fig. S7, see Additional file 1: Supplemental Analysis).

Ribosomal proteins were among the most strongly conserved core OGs, with all but three observed in at least 80% of core genomes (L25, L36, L34) (Fig. 2d). On the *Escherichia coli* ribosome (PDB: 7K00) [21], L25 and L36 are in close proximity to each other (23 Å) and L25 is in contact with both the 5S rRNA and L16, with the latter directly contacting A and P site tRNAs. L34 is embedded within the 50S subunit, interacting primarily with the 23S rRNA and further away from the tRNAs (34 Å to A and P site tRNAs, 40 Å to E site tRNA) (Fig. 2e). The reduced conservation of these ribosomal proteins is consistent with previous experiments suggesting their non-essentiality, with viable deletion mutants having been generated for L25 and L36 in *E. coli* and *Bacillus subtilis* and for L34 in *B. subtilis* only [22].

Finally, a number of highly conserved core OGs were under-characterized and likely comprise an integral but poorly understood component of bacterial genomes. 72 OGs corresponding to y-genes or annotated with the “S: Function unknown” category were found in > 50% of core genomes, of which 28 had Uniprot annotation level ≤ 3 (Fig. 2f, Additional file 1: Fig. S6b-c) and four had entirely no functional annotation: *yihY*, *yicG*, *yicC*, and *yeaQ* (Additional file 1: Table S1). Overall, we find that while the allocation of core genes to functional categories is similar across species, core genomes appear highly diverse at the level of individual OGs, with several under-characterized OGs consistently present across most species. Annotations for conserved OGs are available in Additional file 5.

### Reconstruction of the last bacterial common ancestor core genome

We next used these 183 core genomes to reconstruct the core genome of the LBCA based on the species-level Web of Life subtree extracted earlier. Reconstruction was carried out using Count [23] under asymmetric Wagner parsimony [13], using the core OG by species presence/absence table as input. Briefly, given a set of genes for each modern species, this approach assigns genes to each ancestral species such that the number of gene gain/loss events is minimized, where the relative penalty for a gain vs. loss event can be adjusted. As the appropriate gain/loss penalty ratio is not known for core genomes, multiple reconstructions were conducted with different ratios ranging from 0.1 to 2.0. OGs recovered at smaller ratios (and consequently smaller, more stringently defined ancestral core genomes) were treated as more likely to be present in ancestral core genomes, similar to a previous interpretation of the gain/loss ratio [24].

**Fig. 3:**
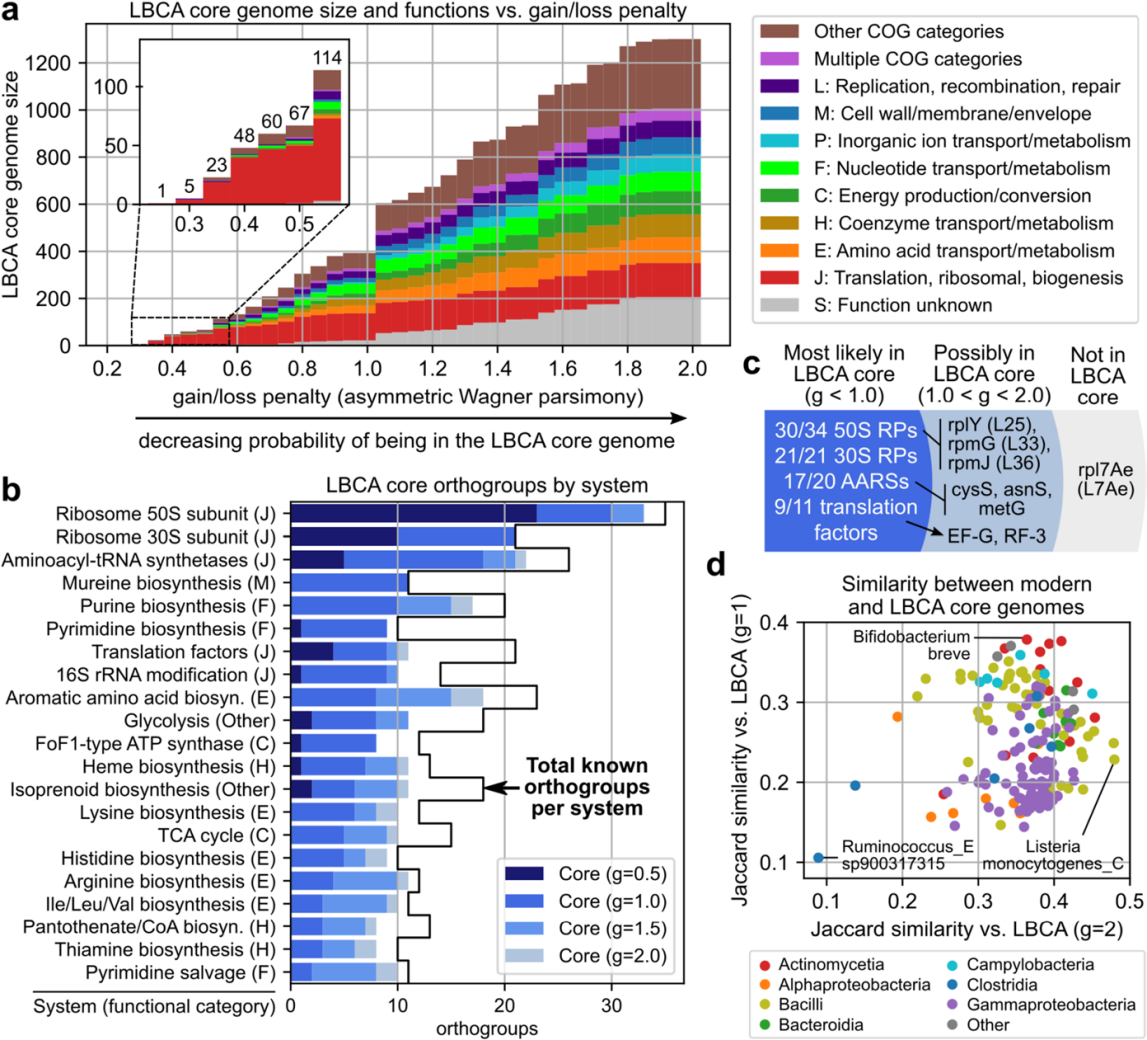
Distribution of gene functions in the core genome of the last bacterial common ancestor. **a** Size and functions of the last bacterial common ancestor (LBCA) core genome as the gain/loss penalty ratio (*g*) is increased during ancestral reconstruction through asymmetric Wagner parsimony. Functional categories comprising at least 5% of the core genome at* g* = 2.0 are shown. **b** Distribution of LBCA core orthogroups by biological system and minimum gain/loss penalty to be observed. Systems with at least 10 orthogroups of which at least 50% are present in the LBCA core genome at* g* = 2.0 and at least one at* g* = 1.0 are shown. **c** Distribution of LBCA core orthogroups related to ribosomal proteins (RPs), aminoacyl-tRNA synthetases (AARSs), and translation factors. **d** Jaccard similarity between modern and LBCA core genomes at* g* = 1.0 and* g* = 2.0, by species phylogenetic class. The core genomes least and most similar to that of the LBCA for* g* = 1.0 and* g* = 2.0 are labeled

The size of the LBCA core genome grew steadily with the gain/loss penalty ratio (*g*), with the minimum ratio at which an OG was observed in the LBCA core genome correlated with the fraction of modern core genomes with the OG (*p* =  − 0.814, Additional file 1: Fig. S8a). A large jump in core genome size was observed between *g* = 1.0 and *g* = 1.05 as the model switches from preferring gain events to loss events (Fig. 3a). The first LBCA core OG was observed at *g* = 0.25 (*rplT*, 50S ribosomal protein L20), and the size of the LBCA core genome exceeded 100 OGs at *g* = 0.55 and 1000 OGs at *g* = 1.55. OGs recovered at the smallest ratios were again mostly translation-associated, which remained the most common functional category for all ratio values (excluding S: function unknown). Other functional categories representing at least 5% of the LBCA core genome at *g* = 2.0 were primarily metabolic, involving amino acid (E), coenzyme (H), energy (C), nucleotide (F), or inorganic ion (P) metabolism. Other highly represented categories included “M: Cell wall, membrane, envelope biogenesis” and “L: Replication, recombination and repair”. Finally, the LBCA core genomes were also compared with those reconstructed using GLOOME, which applies a maximum likelihood approach to reconstruction rather than parsimony-based optimization [25]. Under default settings (fixed gain/loss ratio, gamma-distributed rates of variation), LBCA core genomes from GLOOME were consistent with parsimony-based core genomes in terms of both overall gene content and thresholds for individual genes (i.e., minimum gain/loss ratio for parsimony models vs. posterior probability for GLOOME), and OGs recovered at *g* = 1.0 with the parsimony-based approach had mean and minimum GLOOME posterior probabilities of 0.9997 and 0.9921, respectively (Additional file 1: Fig. S9, see Additional file 1: Supplemental Analysis).

### Functional analysis suggests a versatile LBCA core genome

Annotation of these OGs with biological systems from the 2020 COG database [26] revealed that certain systems were nearly entirely present in the LBCA core genome. Among systems with at least 10 known OGs, 21 had over half of their known OGs present in the LBCA core genome at *g* = 2.0 and at least one at *g* = 1.0, and were again primarily translation-associated or metabolic systems, especially those related to nucleotide or amino acid biosynthesis (Fig. 3b, Additional file 1: Fig. S8b). Eight such systems had > 90% of their known OGs present in the LBCA core at *g* = 2.0: Mureine biosynthesis, Ribosome 30S subunit, Ribosome 50S subunit, Arginine biosynthesis, Pyrimidine salvage, Ile/Leu/Val biosynthesis, Pyrimidine biosynthesis, and Histidine biosynthesis. Eight systems also retained > 80% of their OGs at the more stringent *g* = 1.0 threshold: Mureine biosynthesis, Ribosome 30S subunit, Ribosome 50S subunit, Pyrimidine biosynthesis, FoF1-type ATP synthase, 16S rRNA modification, Translation factors, and Aminoacyl-tRNA synthetases. LBCA OG thresholds and annotations are available in Additional file 5.

We examined four systems associated with translation with many OGs present in the LBCA core at stringent gain/loss penalty ratios: 50S ribosomal proteins, 30S ribosomal proteins, aminoacyl-tRNA synthetases (AARSs), and translation factors. Most OGs in these systems are present at* g* = 1.0 and highly likely to be in the LBCA core genome: 30/34 50S ribosomal proteins, 21/21 30S ribosomal proteins, 17/20 AARSs, and 9/11 bacterial translation factors (Fig. 3c). All but one of the nine OGs missing at *g* = 1.0 were present at *g* = 2.0, and only *rpl7Ae* (ribosomal protein L7Ae) was missing entirely. The eight marginal core OGs consisted of 50S ribosomal proteins *rplY* (L25), *rpmG* (L33), and *rpmJ* (L36) observed at *g* = 1.05 (Additional file 1: Fig. S10a); AARSs *cysS* (CysRS, observed at *g* = 1.1), *asnS* (AsnRS, *g* = 1.3) and *metG* (MetRS, *g* = 1.6) (Additional file 1: Fig. S10b); and translation factors *fusA* (EF-G, *g* = 1.05) and *prfC* (RF3, *g* = 1.6) (Additional file 1: Fig. S10c). Most of these OGs are known to be non-essential, missing in some bacterial and/or archaeal species, and/or have known compensatory paralogs or pathways (Additional file 1: Table S2). Only *metG* and *fusA* have no previous studies that suggest their absence from LBCA but are known to have complex evolutionary histories resulting in highly diverse sequences [27, 28] and potentially incomplete coverage by a single OG as recoverable by eggNOG. Nonetheless, ancestral reconstruction from core genomes seems to suggest that most, if not all of these translation-related genes traditionally thought of as universal are indeed likely to be present in not just individual LBCA genomes but in the LBCA species-wide core genome.

Analyzing OGs assigned to the “S: Function unknown” category or corresponding to y-genes revealed 108 potentially under-characterized OGs in the LBCA core genome at* g* = 2.0, 10 of which were present at *g* = 1.0 (Additional file 1: Fig. S8c). All but one of the 10 under-characterized core OGs at *g* = 1.0 was observed in at least 50% of modern core genomes and were among the highly conserved under-characterized core OGs reported earlier (*yqxC* observed in 33%). Finally, comparisons between the LBCA and modern core genomes finds the LBCA to be genetically distinct from all 183 modern species in this analysis. Jaccard similarities between the LBCA core genome (at either *g* = 1.0 or *g* = 2.0) and modern core genomes did not exceed 0.5, though core genomes from certain phylogenetic classes were more similar to that of the LBCA than others (Fig. 3d). Campylobacteria had the highest median similarity at *g* = 2.0, Bacteroidia at *g* = 1.0, and Actinomycetia was the 2nd most similar for both thresholds.

### Comparison against minimal organism JCVI-Syn3A suggests that the LBCA core genome alone is not sufficient for viability

**Fig. 4:**
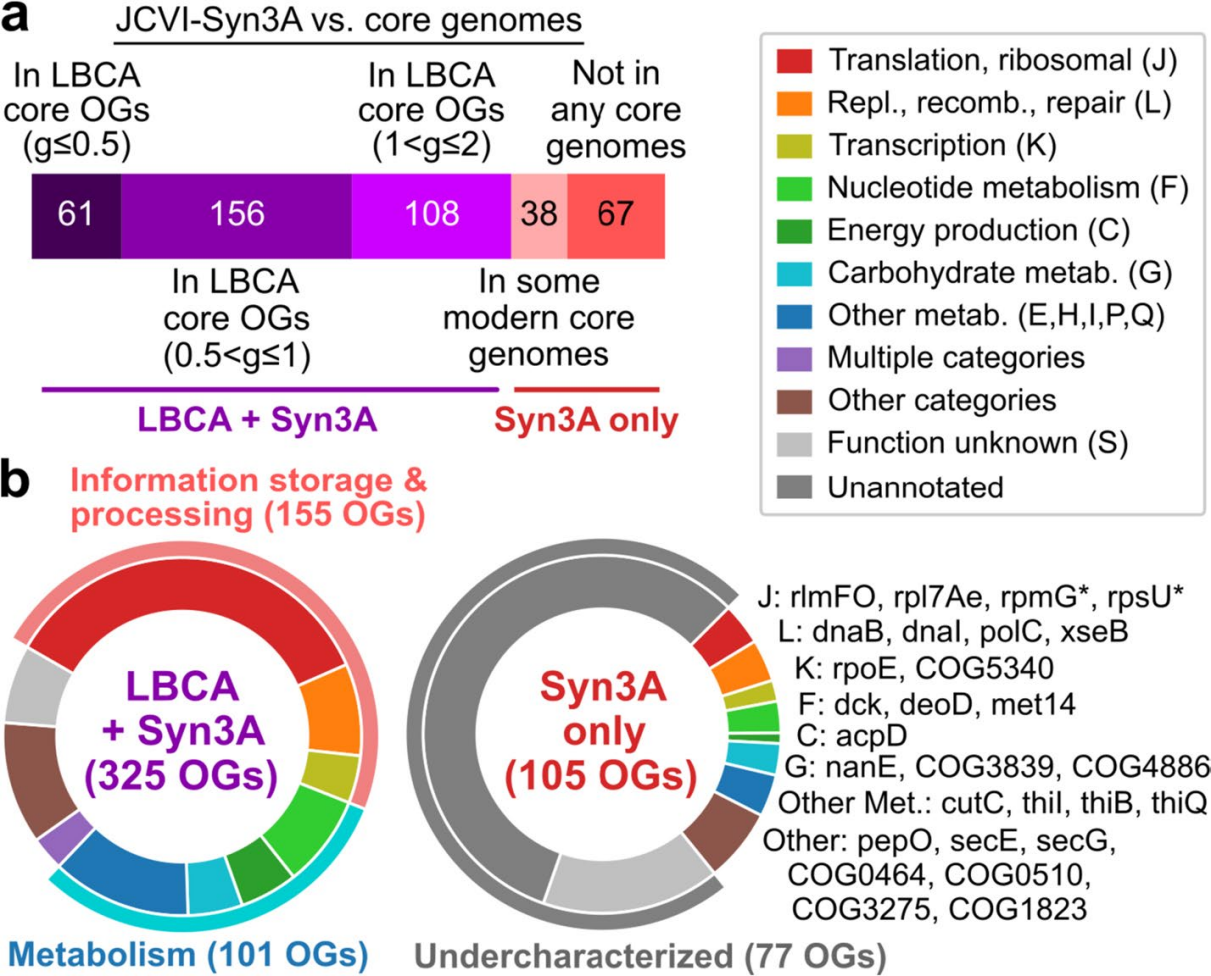
Similarities between the LBCA core genome and the genome of minimal organism JCVI-Syn3A. **a** Distribution of Syn3A orthogroups (OGs) in the LBCA and modern core genomes. Modern core genomes refer to those initially constructed for 183 bacterial species. **b** Distribution of functional categories among OGs shared by the LBCA core genome (*g* = 2.0) and Syn3A, and those in Syn3A only. Starred OGs refer to alternate variants of OGs present in the LBCA core genome

To explore how close the LBCA core genome is to a minimal set of genes required for life, we compared its gene content with the genome of JCVI-syn3A (Syn3A), a synthetic minimal organism of 493 genes (452 protein-coding) derived from *Mycoplasma mycoides capri* (GenBank: CP016816.2) [29]. Annotation with eggNOG-emapper was able to identify 430 unique OGs in Syn3A. 325 of these OGs (76%) were present in the LBCA core genome at its largest extent at *g* = 2.0, with a majority recovered at *g* = 1.0 (50%, 217/430) (Additional file 1: Fig. S11a, Additional file 5). Conversely, nearly all LBCA core OGs present at the most stringent penalty ratios are also present in Syn3A, but the fraction of LBCA OGs in Syn3A drops to 55% (217/393) by *g* = 1.0 and 25% (325/1301) by *g* = 2.0 (Additional file 1: Fig. S11a). An additional 38 Syn3A OGs were observed in at least one of the 183 modern core genomes but not in the LBCA core genome, and the remaining 67 OGs were not observed in any core genome (Fig. 4a).

Comparing the LBCA core genome, the Syn3A genome, and the core genome of related small genome species *Mycoplasmoides pneuomiae* also finds extensive but not complete overlap between the three gene sets (Additional file 1: Fig. S11b). 13 OGs were in both the core genome of *M. pneumoniae* and the Syn3A genome but not in the LBCA core genome, while 92 Syn3A OGs were missing from both core genomes. Functional annotation found most of the 325 OGs shared by LBCA and Syn3A to be well-characterized and primarily ribosomal or metabolic, while the 105 non-LBCA OGs in Syn3A were mostly under-characterized proteins (77/105) with the remaining 28 OGs being of diverse functions (Fig. 4b). These results suggest that most of the genes in the minimal organism Syn3A likely descended from the LBCA core genome, but given the requirement of additional non-core genes for viability, also suggest that the LBCA core genome alone may be insufficient for survival.

### Pathway analysis suggests a highly metabolically self-sufficient LBCA core genome

**Fig. 5:**
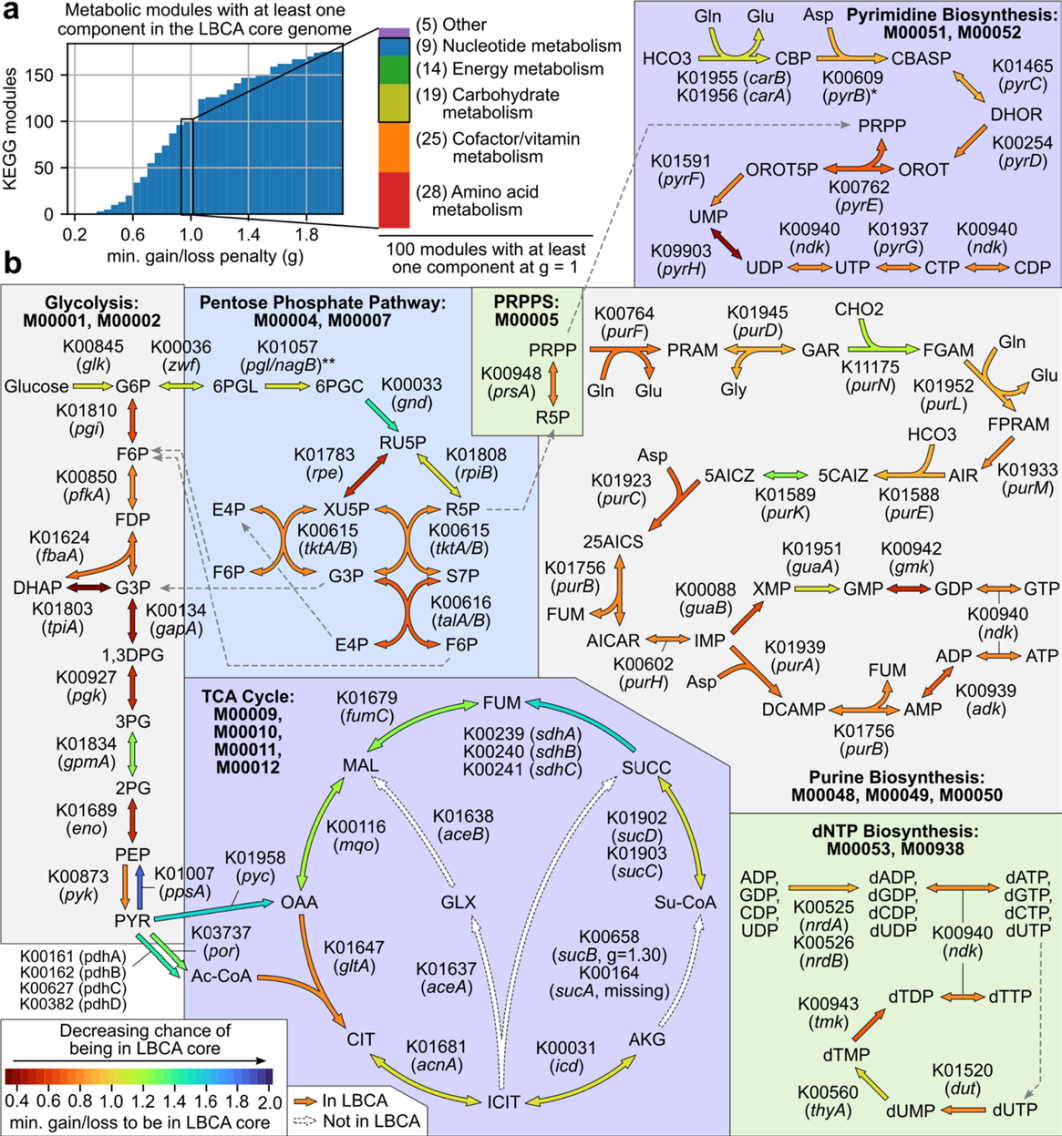
Metabolic modules involving central carbon metabolism or nucleotide metabolism represented in the LBCA core genome. **a** Distribution of KEGG modules with at least one related orthogroup present in the LBCA core genome for varying gain/loss penalty thresholds (*g*). Module categories at the* g* = 1.0 threshold are shown, with visualized modules boxed. **b** Individual reactions in the LBCA core genome related to modules involving central carbon metabolism or nucleotide metabolism. Each reaction is labeled with the corresponding KEGG orthogroup(s) and gene name(s) and is colored by the minimum penalty ratio at which the corresponding orthogroup is present in the LBCA core (or maximum of ratios if multiple orthogroups are required). Missing reactions are colored white. Dashed gray arrows connect identical metabolites. For the starred reaction corresponding to K00609 (*pyrB*), the regulatory subunit K00610 (*pyrI*) is missing from the LBCA core genome but is not required for catalytic activity. For the double-starred reaction corresponding to K01057 (*pgl*), the closest COG orthogroup COG0363 represents sequences from both *pgl* and *nagB*

To examine the metabolic capabilities of the LBCA core genome at the pathway level, COG orthogroups were mapped to KEGG orthogroups [30] through eggNOG-emapper annotations and then to KEGG modules (Additional file 6). 100 and 175 KEGG modules had at least one reaction active based on KEGG orthogroups present at *g* = 1.0 and *g* = 2.0, respectively, primarily from five categories: amino acid metabolism, cofactor/vitamin metabolism, carbohydrate metabolism, energy metabolism, and nucleotide metabolism (Fig. 5a). Reconstruction of modules in the last three categories revealed that much of central carbon metabolism and biosynthetic pathways to all major nucleotides were largely intact even at *g* = 1.0 (Fig. 5b).

Just three components in central carbon metabolism were missing from the LBCA core genome: (1) *sucA*, the E1 component of the α-ketoglutarate dehydrogenase complex in the TCA cycle, (2) *aceA*, isocitrate lyase in the glyoxylate cycle, and (3) *aceB*, malate synthase in the glyoxylate cycle. Several reactions were only active at high g: two mechanisms of generating acetyl-CoA from pyruvate (pyruvate dehydrogenase complex and pyruvate-ferredoxin oxidoreductase) and the second half of the TCA cycle (succinate to oxaloacetate) were only active at *g* = 1.2 or higher. In nucleotide metabolism, de novo biosynthetic pathways to all nucleotides (A,C,G,U) and deoxyribonucleotides (A,C,G,T,U) were active in the LBCA core genome with most reactions present at *g* = 1.0. The only missing OG was *pyrI*, the regulatory subunit of aspartate carbamoyltransferase (the catalytic subunit *pyrB* is independently active [31]).

**Fig. 6:**
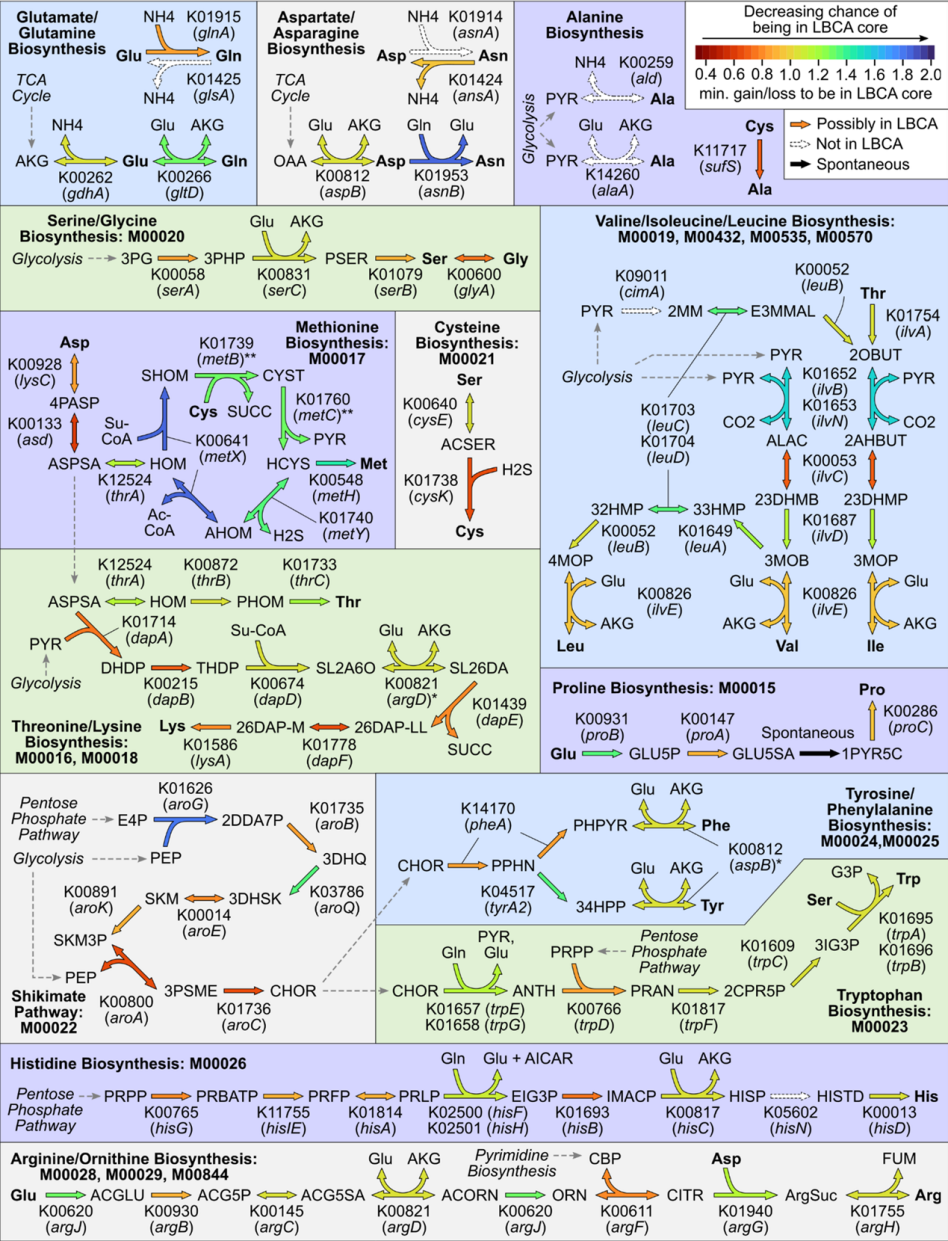
Metabolic modules involving amino acid biosynthesis represented in the LBCA core genome. Each reaction is labeled with the corresponding KEGG orthogroup(s) and gene name(s) and is colored by the minimum penalty ratio at which the corresponding orthogroup is present in the LBCA core genome (or maximum of ratios if multiple orthogroups are required). Missing reactions are colored white and spontaneous reactions are colored black. Amino acid inputs and outputs are bold. The biosynthesis pathways of alanine, aspartate, asparagine, glutamate, and glutamine do not have specific KEGG modules and have been added separately. Starred reactions indicate reactions that can be catalyzed by enzymes in unrelated pathways as the standard enzyme is missing from LBCA: In Lysine biosynthesis, K00821 (*argD*) can catalyze SL2A60 → SL26DA in place of K14267 (*dapC*), and in Tyrosine/Phenylalanine biosynthesis, K00812 (*aspB*) can catalyze the final steps in both pathways in place of K00832 (*tyrB*). For double-starred reactions corresponding to *metB* and *metC*, the COG orthogroup COG0626 present at* g* = 1.3 represents sequences from both genes

A similar reconstruction of amino acid metabolism finds de novo biosynthetic pathways to all 20 amino acids to be mostly intact, with most reactions present at *g* = 1.2 and no more than one reaction missing per pathway (Fig. 6). Six components were missing from the LBCA core genome: (1) *glsA*, glutaminase; (2) *asnA*, aspartate ammonia ligase; (3) *ald*, alanine dehydrogenase; (4) *alaA*, alanine transaminase; (5) *cimA*, citramalate synthase; and (6) *hisN*, histidinol phosphatase. Biosynthetic pathways towards lysine, phenylalanine, and tyrosine also relied on promiscuous aminotransferase activity from other pathways as annotated on KEGG (*argD* and *aspB* in place of *dapC* and *tyrB*, respectively). All but one of these absences either did not preclude the biosynthesis of their corresponding amino acid or could be compensated by other pathways. Orthogroups *glsA* and *asnA* are not required in the biosynthesis of glutamate/glutamine and aspartate/asparagine from TCA cycle intermediates. Alanine could be generated from cysteine via *sufS* in the absence of *ald* and *alaA*, and the isoleucine precursor 2-oxobutanoate could be generated from threonine via *ilvA* in the absence of *cimA*. The absence of *hisN* did not have a clear compensatory pathway, though it is possible that the missing reaction could be catalyzed by another phosphatase or that the LBCA core genome may have had a bifunctional *hisB* capable of carrying out the reaction as observed in Enterobacteria [32].

## Discussion

The vast amount of publicly available genomic data has enabled the reconstruction of evolutionary histories spanning entire domains of life, but has also opened new questions regarding the appropriate structure of such phylogenies and the nature of the ancestors at their roots. With a pangenomic perspective compatible with competing hypotheses on universal ancestry, we reconstructed and characterized the core genome of the LBCA from the pangenomes of 183 species with three tools: (1) pangenome construction by sequence clustering, (2) core gene identification through a novel model for estimating gene frequencies and genome-specific gene recovery rates, and (3) ancestral genome reconstruction with asymmetric Wagner parsimony. We find that the 183 modern core genomes vary significantly at the level of individual genes but similarly allocate genes to different functional categories, and that the LBCA core genome was versatile with many systems related to translation machinery and biosynthetic pathways fully intact.

We first addressed the issue of sequencing artifacts and other errors leading to genes being lost during pangenome construction and confounding the identification of core genes. Rather than adopting an arbitrary cutoff of allowing core genes to be missing from a certain percentage of genomes as is common in previous studies, we estimated the underlying true gene frequencies by modeling gene presence/absence calls in observed pangenomes as Bernoulli random variables and applying maximum likelihood estimation. Applied to the 183 pangenomes, several results support the robustness and reliability of this approach. Estimated genome-specific recovery rates were correlated with existing measures of genome assembly quality and especially with CheckM’s completeness metric. Estimated gene frequencies were also robust to subsampling, were consistent with the existence of true core genomes, followed a consistent distribution shape, and yielded core genomes of similar size to those from previous studies. These results suggest that gene frequency estimation can identify core genes in a manner more faithful to their original definition. More generally, this approach of decomposing presence/absence matrices into feature frequencies and sample recovery rates may inform future analyses of other types of binary biological data on the true distribution of observed features and sample quality.

A comparison of the 183 core genomes defined using estimated frequencies revealed that bacterial species allocate similar fractions of their core genes to specific functional categories, similar to what was previously observed for 12 pathogens [12]. In contrast, core genomes differed greatly at the gene-level with no single OG observed in all 183 core genomes, consistent with previous observations that biochemical functions rather than individual genes tend to be conserved [33]. The few genes that were nearly universally conserved across the core genomes were mostly ribosomal proteins, resembling the set of approximately 30 genes that smaller-scale studies reported present in all genomes [34, 35]. We also identified 28 under-characterized genes that were strongly conserved across core genomes. These genes are ideal candidates for experimental characterization to expand the current understanding of bacterial biology, as any individual bacterial strain is likely to carry them.

We next integrated our core genomes with the Web of Life phylogeny [2] and applied asymmetric Wagner parsimony to generate multiple reconstructions of the LBCA core genome, based on different assumptions regarding the gain/loss ratio or relative tendency towards gene gain vs. loss events along the phylogenetic tree. Even under strict assumptions yielding smaller ancestral core genomes (*g* = 1.0), the LBCA core genome was functionally versatile with many biological systems fully or mostly intact, especially translation machinery. Just 4/56 ribosomal proteins were not present at *g* = 1.0: L25, L33, and L36 have known paralogs and have previously been found to be missing in some genomes [22, 36] and correspondingly resulted in their prediction to be in the LBCA core at the margin (*g* = 1.05), while L7ae is typically associated with archaea [37] and was correctly predicted to be missing. Similarly, just 3/20 aminoacyl-tRNA synthetases were not present at *g* = 1.0. Lack of CysRS and AsnRS can be compensated through alternate pathways [38, 39] and are known to be missing in some genomes [34]. However, no evidence currently exists for MetRS’s absence from the LBCA, and given its critical role in the initiation of translation it is possible that this result is an artifact of clustering and annotation. Finally, 2/11 translation factors were not present at *g* = 1.0: EF-G, which is believed to ancient but has known paralogs [40] was also recovered at the margin (*g* = 1.05), while RF-3’s much later recovery (*g* = 1.6) is consistent with a previous prediction that RF-3 is a post-LBCA offshoot of EF-G [41]. These results confirm many previous predictions regarding the presence of fundamental biological systems in ancestral bacteria to the stronger standard of being present in the LBCA core genome.

However, a comparison with the genome of JCVI-Syn3A suggests that the LBCA core genome alone may not be viable. While the two gene sets overlap significantly, 25% of the genes in Syn3A are still missing from the LBCA core even with relaxed assumptions (*g* = 2.0), most of which were under-characterized. Previous multi-strain studies on gene essentiality for *Pseudomonas aeruginosa* [42] and *Streptococcus pyogenes* [43] have found core genomes to only capture a fraction of any individual strain’s essential gene set, and in vivo genome reduction efforts have yielded organisms with significantly different sets of essential genes even with similar starting strains [44, 45]. Consequently, it is likely that core genomes, including that of the LBCA, must be supplemented with accessory genes to yield a functioning organism, and is consistent with claims that core genes and essential genes are not synonymous concepts [44].

Metabolic reconstruction of the LBCA core genome provides further evidence in support of its versatility. Much of central carbon metabolism and de novo biosynthetic pathways to all major nucleotides and amino acids are present at *g* = 1.0 and fully intact at *g* = 2.0. As observed in a previous metabolic analysis of the LBCA predicting its ability to synthesize all 4 DNA bases, 4 RNA bases, and 20 amino acids [3], this pangenomic analysis provides further evidence for a prototrophic LBCA through the identification of individual reaction-specific orthogroups that are predicted to be universally conserved within the LBCA species. It has also been predicted that all but four amino acids could be synthesized by the ancestor of all three domains of life [46]; three of these (lysine, phenylalanine, tyrosine) could be synthesized by our predicted LBCA core genome when assuming promiscuous activity from aminotransferases present in the other amino acid biosynthetic pathways predicted to be active in the universal ancestor [46].

While most genes we predicted missing from the LBCA core genome did not preclude the biosynthesis of essential metabolites, the most impactful was the lack of *sucA*, the E1 component of the α-ketoglutarate dehydrogenase complex (AKGDH). The presence of *sucB*, the E2 component, without *sucA* has been observed in *Mycobacterium tuberculosis*, which only carries an E2-like gene and exhibits no AKGDH activity [47]. More broadly, the absence of AKGDH, as well as other energy metabolism complexes (i.e., succinate dehydrogenase, pyruvate dehydrogenase) only being detected for large gain/loss ratios, may be a result of the outsized diversity observed for such modular complexes [48] confounding ancestor reconstruction. Nonetheless, it is worth noting that the absence of these genes from the LBCA core genome does not mean that the LBCA was entirely unable to catalyze the corresponding reactions. Rather, the missing genes may have instead been part of the LBCA accessory genome, allowing some LBCA strains to carry out the reactions while others adapted alternate genetic and metabolic solutions for survival. Such intraspecies diversity in the LBCA likely contributed to the emergence of modern interspecies diversity.

The core metabolism of the LBCA also provides some clues into its contemporary environment. The absence of a direct pathway from pyruvate to alanine is compensated by *sufS* enabling the conversion of cysteine to alanine, consistent with previous hypotheses highlighting the prevalence of sulfur chemistry in early life [49]. Comparing alternate amino acid biosynthesis pathways, the LBCA core genome contained the thermodynamically favorable pathway over the cofactor efficient pathway in all five acyl-CoA dependent pathways [50], which suggests that cofactor efficiency may not have been a major driver of selection even in the anaerobic environment of ancient bacteria. Similarly, the absence of the glyoxylate cycle, which provides a net anaplerotic reaction required for growth with certain organic acids such as acetate as sole carbon sources [51], suggests that such environments may have been too rare to establish glyoxylate cycle enzymes as part of the LBCA core genome.

## Conclusions

Overall, the integration of pangenomics and phylogenetics enabled by the current scale of public genomic data offers a new, flexible perspective for reconstructing and characterizing ancestral species. Our analysis of 54,085 genomes and 183 species suggests that the bacterial ancestor was genetically and metabolically versatile, confirming many previous predictions regarding genes in the LBCA to the higher standard of being in its core genome. While this work relies on the Web of Life phylogenetic tree, future analyses may consider constructing phylogenies directly from core genomes to reduce the confounding effects of horizontal gene transfer. We also note that due to the distribution of species for which sufficient genomes were available for pangenome construction, this analysis likely overrepresents aerobes and proteobacteria which can bias the reconstructed LBCA regardless of computational technique. As more genome sequences are generated for a greater diversity of species, future analyses may improve upon both the coverage and scope of this work to reconstruct more distant ancestral organisms with greater confidence. Finally, expanding the analysis from core genomes to full pangenomes would shed light on whether a species’ weakly maintained accessory genes are ephemeral at an evolutionary time scale or are a persistent and co-evolving feature of the species. Ultimately, as observed biological variation appears multimodal and suggests the existence of species [52], efforts to chart the evolutionary history of life will benefit from employing both pangenomic and phylogenetic methods that offer complementary perspectives on intra- and inter-species genetic diversity.

## Methods

### Pangenome construction

Genomes from the Web of Life collection [2] were filtered based on the following criteria: (1) GTDB species classification (release 202) is available [15], (2) CheckM contamination < 10% and completeness > 80% [14], (3) number of contigs is within three times the median number of contigs for all assemblies for the genome’s species, and (4) total assembly length is within three standard deviations from the mean of all assemblies for the genome’s species. One hundred eighty-three bacterial species defined by GTDB were identified with at least 50 genomes passing all criteria, totalling 54,085 genomes. NCBI accession IDs, GTDB phylogenetic classifications, and genome quality metrics for selected genomes are available in Additional file 2.

Open reading frames for each genome were identified using Prodigal v2.6.3 with parameters “-c,” “-m,” “-g,” “11,” “-p,” “single,” and “-q” [16], based on those used in by the Prokka annotation platform [53]. For each species, genes and protein sequence variants were identified using the approach previously described [12]. Briefly, protein sequences were clustered using CD-HIT v4.6 with minimum identity 80% and minimum alignment length 80% [17] and clusters were treated as genes.

### Species-level phylogeny construction

A species-level phylogeny for the 183 selected species was extracted from the genome-level Web of Life phylogeny as follows. For each species, the most recent common ancestor (MRCA) of its selected genomes was identified. For 19 species where the MRCA contained children from multiple GTDB species (i.e., was not monophyletic), an alternate MRCA representative was identified by computing for each child node its completeness (fraction of the species’ selected genomes present among the node’s children) and purity (fraction of the node’s children that are of that species), and selecting the node with the highest harmonic mean between completeness and purity. Each MRCA was extended by the median distance to its species’ selected genomes to model a modern representative of the species. Finally, the phylogeny was pruned such that the only child nodes are the 183 species representatives, and internal nodes with only 1 child were collapsed by adding distances to yield a binary phylogenetic tree. The final species-level tree is available in Additional file 3, and representative node assignments in Additional file 2.

### Gene frequency estimation

True gene frequencies were estimated by modeling the log-likelihood (LL) of an observed gene presence/absence matrix **X** as follows:

$$LL\left(\mathbf{X},\mathbf{p},\mathbf{q}\right)=\sum\limits_{i=1}^{m}\sum\limits_{j=1}^{n}{x}_{ij}log\left({p}_{i}{q}_{j}\right)+\left(1-{x}_{ij}\right)log\left(1-{p}_{i}{q}_{j}\right)$$

where *x*_*ij*_ = 1 if gene *i* was observed in genome *j* or 0 otherwise, *p*_*i*_ is the true frequency of gene *i* in the genome collection, *q*_*j*_ is the gene recovery rate of genome *j*, *m* is the total number of genes, and *n* is the total number of genomes. Variables **p** and **q** were estimated by maximizing the LL using SciPy [54] with the L-BFGS-B method, with bounds [10^−8^, 1 − 10^−8^] for all variables and initial guesses 0.99 for all *q*_*j*_ and respective observed gene frequencies (fraction of genomes with the gene) for *p*_*i*_. Estimation was limited to genes with observed frequency > 10%, and maximization was accelerated using exact gradients:

$$\frac{\partial LL}{\partial {p}_{k}}=\sum_{j=1}^{n}\frac{{x}_{kj}}{{p}_{k}}-\frac{\left(1-{x}_{kj}\right){q}_{j}}{1-{p}_{k}{q}_{j}},\frac{\partial LL}{\partial {q}_{k}}=\sum_{i=1}^{m}\frac{{x}_{ik}}{{q}_{k}}-\frac{\left(1-{x}_{ik}\right){p}_{i}}{1-{p}_{i}{q}_{k}}$$

### Benchmarking gene frequency estimation

Estimated gene recovery rates were compared to quality metrics available on GTDB. For computing modes and fitting gene frequency distributions, distributions were discretized into bins of size 1/number of genomes for each species. For fitting frequency distributions, distributions were first reduced to those with observed frequency > 90% (potential core genes). Both observed and estimated distributions were fit to a power model [12] or exponential model [55] by fitting their corresponding cumulative distributions (Additional file 1: Fig. S3c), where *P*(*x*) is the number of genes with frequency *x* and *F*(*x*) is the number of genes with frequency ≤ *x*. Models were fit to cumulative distributions rather than directly to frequency distributions to avoid fitting data spanning multiple orders of magnitude. Parameters were estimated using the SciPy curve_fit routine [54], with observed *F*(*x*) scaled to maximum value 1 and with initial guesses for (k,c,a) of (1,1,2) for the power model and (1,1,1) for the exponential model. All parameters were bounded positive except for the power model “a” parameter which was bounded a > 1. Parameters from fitting *F*(*x*) were applied directly to the corresponding *P*(*x*) function and quality of fit was evaluated through *R*^2^ and mean absolute error (MAE).

### Core genome identification and annotation

For each species, the core genome was defined as all genes with estimated frequency > 99.99%. The most common sequence variant of each gene in each species was annotated using eggNOG-emapper v2.1.6–43 [20] to map each gene to COG functional categories and orthogroup (OG), using the highest depth annotations. All cross-species analyses were conducted at the OG level. OG gene names were assigned by comparing annotations from eggNOG-emapper, the 2020 COG database [26], and UniprotKB [56]. Under-characterized OGs were identified by first filtering for OGs assigned to the category “S: Function unknown” or corresponding to a gene name starting with “y”, then identifying those with low UniprotKB annotation scores as of September 7, 2022.

### LBCA core genome reconstruction, analysis, and comparison with JCVI-Syn3A

Each species’ node in the species-level phylogeny was assigned the species’ core genome OGs as previously identified. OG content of internal ancestral nodes in the phylogeny was estimated using Count [23] under Asymmetric Wagner parsimony [13] for gain/loss penalty ratios ranging from 0.1 to 2.0 in 0.05 steps. The last bacterial common ancestor (LBCA) core genome for a given penalty ratio was taken as the set of OGs predicted for the root node of the phylogeny. COG functional categories, OG names, and under-characterized OGs were assigned the same as for modern core genomes. OG systems were assigned from the 2020 COG database [26].

For system case studies, the set of 55 bacterial 50S and 30S ribosomal proteins was taken from Yutin, et.al. 2012 [36], excluding proteins S22 and S31e which did not have COG database IDs. Aminoacyl-tRNA synthetases were limited to those corresponding to the 20 canonical amino acids and classifications were taken from Gomez and Ibba, 2020 [39]. Translation factors were taken directly from the 2020 COG database after filtering out eukaryotic proteins. Finally, for comparison with JCVI-syn3A, the JCVI-syn3A assembly with predicted ORFs (GenBank: CP016816.2) was similarly annotated with eggNOG-emapper to assign proteins to OGs.

### LBCA core metabolism reconstruction

LBCA core OGs were mapped to KEGG orthogroups [30] through eggNOG-emapper annotations, by first identifying all sequences annotated with a given OG and taking the most common KEGG annotation among those sequences. LBCA core KEGG orthogroups were then mapped to KEGG modules by evaluating KEGG module definitions as logical expressions. KEGG modules with at least one KEGG orthogroup corresponding to an OG present in the LBCA core at *g* = 1.0 were visualized. Multi-enzyme reactions were treated as active if all necessary orthogroups were present. Missing reactions were manually verified for alternate pathways on KEGG, other orthogroups capable of catalyzing the reactions, and other COG orthogroups that could be mapped to the missing KEGG orthogroup based on curating eggNOG text annotations.

### Supplementary Information

**Additional file 1: Supplemental Analysis, Figures, and Tables. Figure S1.** Properties of selected genomes by species and phylogenetic class. **Figure S2.** Analysis of estimated gene recovery rates and gene frequencies with respect to genome quality and robustness to random genome sampling. **Figure S3.** Analysis of estimated gene frequency distributions. **Figure S4.** Comparisons between three definitions of core genome across 183 species. **Figure S5.** Principal component analysis of core genome allocation of genes to functional categories. **Figure S6.** Survey of orthogroups frequently observed in core genomes. **Figure S7.** Sensitivity of conserved orthogroups to core genome frequency threshold. **Figure S8.** Additional analyses of the LBCA core genome gene content. **Figure S9.** Comparison between LBCA core genomes reconstructed under asymmetric Wagner parsimony with Count vs. maximum likelihood with GLOOME. **Figure S10.** Distribution of LBCA core orthogroups related to ribosomal proteins, aminoacyl-tRNA synthetases, and translation factors. **Figure S11.** Breakdown of gene overlap between the LBCA core genome and genome of minimal organism JCVI-Syn3A. **Table S1.** 28 under-characterized orthogroups frequently observed in core genomes. **Table S2.** Translation-associated orthogroups from three systems only present in the LBCA core genome when reconstructed with gain/loss penalties (g) above 1.0.

**Additional file 2: DatasetS1.** Accession IDs, GTDB phylogenetic classifications, and assembly quality metrics for all genomes used in this study. Also includes node IDs for species’ representative nodes in the phylogenetic tree used for ancestral reconstruction.

**Additional file 3: DatasetS2.** Species phylogenetic tree used for ancestral reconstruction.

**Additional file 4: DatasetS3.** Results of benchmarking experiments for estimating true gene frequencies and genome-specific gene recovery rates from pangenomes.

**Additional file 5: DatasetS4.** Orthogroup analysis of modern core genomes, the LBCA core genome, and JCVI-Syn3A. Includes estimated orthogroup frequencies, annotations, functional categories, and LBCA prediction thresholds (gain/loss ratios from parsimony models, posterior probabilities from GLOOME).

**Additional file 6: DatasetS5.** Metabolic analysis of the LBCA core genome. Includes mappings between COG and KEGG orthogroups, active KEGG modules in the LBCA core genome, and metabolite abbreviations for LBCA metabolic pathways.

**Additional file 7. **Review history.

## Data Availability

The genomes analyzed during the current study are publicly available through NCBI on RefSeq (https://www.ncbi.nlm.nih.gov/refseq/) or GenBank (https://www.ncbi.nlm.nih.gov/genbank/), with accession IDs provided in Additional file 2. The phylogenetic tree used for ancestral reconstruction is available in Additional file 3.
