# Supplementary material for: Reconstruction of the last bacterial common ancestor from 183 pangenomes reveals a versatile ancient core genome
Source: Genome Biol. 2023 Aug 8;24:183. doi: 10.1186/s13059-023-03028-2 (PMC10411014; doi:10.1186/s13059-023-03028-2)
Supplement: Supplementary file 1 — Additional file 1: Supplemental Analysis, Figures, and Tables. Figure S1. Properties of selected genomes by species and phylogenetic class. Figure S2. Analysis of estimated gene recovery rates and gene frequencies with respect to genome quality and robustness to random genome sampling. Figure S3. Analysis of estimated gene frequency distributions. Figure S4. Comparisons between three definitions of core genome across 183 species. Figure S5. Principal component analysis of core genome allocation of genes to functional categories. Figure S6. Survey of orthogroups frequently observed in core genomes. Figure S7. Sensitivity of conserved orthogroups to core genome frequency threshold. Figure S8. Additional analyses of the LBCA core genome gene content. Figure S9. Comparison between LBCA core genomes reconstructed under asymmetric Wagner parsimony with Count vs. maximum likelihood with GLOOME. Figure S10. Distribution of LBCA core orthogroups related to ribosomal proteins, aminoacyl-tRNA synthetases, and translation factors. Figure S11. Breakdown of gene overlap between the LBCA core genome and genome of minimal organism JCVI-Syn3A. Table S1. 28 under-characterized orthogroups frequently observed in core genomes. Table S2. Translation-associated orthogroups from three systems only present in the LBCA core genome when reconstructed with gain/loss penalties (g) above 1.0. [file 13059_2023_3028_MOESM1_ESM.pdf]

Reconstruction of the last bacterial common ancestor from 183 pangenomes reveals a versatile ancient core genome

Jason C. Hyun[1] and Bernhard O. Palsson[1,2]*

[1] Bioinformatics and Systems Biology Program, University of California, San Diego, La Jolla, CA, USA
[2] Department of Bioengineering, University of California, San Diego, La Jolla, CA, USA.
* Corresponding author
Email: palsson@ucsd.edu (BOP)

Supplemental Analysis, Figures, and Tables

Table of Contents

Supplemental Analysis..2
Supplemental Figures..5
Supplemental Tables..15

Supplemental Analysis

Benchmarking estimation of genome-specific gene recovery rates and true gene frequencies

True gene frequencies (p_i) and genome-specific gene recovery rates (q_j) were estimated simultaneously by maximizing the log-likelihood of the observed presence/absence matrix, where x_{ij} = 1 if gene i was observed in genome j or 0 otherwise, and m and n are the total number of genes and genomes, respectively:

$$LL(\mathbf{X}, \mathbf{p}, \mathbf{q}) = \sum_{i=1}^{m} \sum_{j=1}^{n} x_{ij} log(p_i q_j) + (1 - x_{ij}) log(1 - p_i q_j)$$

Applied to the 183 species' pangenomes, we find that the estimated genome-specific gene recovery rates are correlated with existing metrics of genome assembly quality. The median Spearman correlations across all species between the estimated rates and negative metrics of quality (L50, number of contigs, CheckM strain heterogeneity, CheckM contamination) were negative, while such correlations with positive metrics of quality (N50, CheckM completeness) were positive (Figure S2b). Particularly, the rates were consistently strongly correlated with CheckM completeness (median Spearman correlation 0.522), in line with the intuition that these rates represent catch-all probabilities for whether a given gene in a strain is recovered by each genome's assembly and annotation process. Across the 3,451 genomes with exactly one contig, CheckM completeness and gene recovery rates were strongly correlated (Pearson correlation 0.724, Figure S2c), and 65% of those genomes achieved gene recovery rates above 99.9999% (Figure S2d). These results suggest that estimated gene recovery rates derived from the pangenome matrix alone are consistent with several commonly used metrics of assembly quality.

We find that the estimated gene frequency distributions are robust to sampling and consistently yield frequency peaks at 100%, in line with the existence of core genomes. In bootstrapping experiments of six phylogenetically diverse species where 90% of genomes were randomly sampled and frequencies were re-estimated (10 samplings per species), the lowest Spearman correlation between frequencies from pairs of samplings exceeded 94% for all species and 97% in 3/6 species (Figure S2e). Examining potential core genes (observed frequency > 90%), the modes of estimated frequencies using bins of size 1/number of genomes was 100% for all 183 species, while the modes of observed frequencies was less than 100% in 67/183 (37%) species (Figure S3a-b).

Estimated frequency distributions also had a more consistent functional form than observed frequency distributions. Observed and estimated frequency distributions of potential core genes were fitted to two previously proposed models for gene frequency distributions (Figure S3c, exponential [55] and power function [12]). Across the 183 species, the estimated frequencies more consistently fit the power model than observed frequencies fit either model, with a minimum R^2 of 0.69 compared to 0.00, 0.02, and 0.05 of the other fitting combinations, and a median R^2 of 0.96 compared to 0.82, 0.92, and 0.74 (Figure S3d-e).

Finally, core genomes defined from estimated frequencies were of similar size to those

defined using traditional approaches. Three core genomes were defined for each species as genes having either 1) estimated frequency > 99.99%, 2) observed frequency >99%, or 3) observed frequency >95%. The sizes of estimation-based core genomes were between that of the 99% and 95% observed frequency core genomes and strongly correlated (r = 0.79 against 99% core genomes, r = 0.93 against 95% core genomes, Figure S4a-b). Gene content was more variable between definitions, with median Jaccard similarities between estimation-based core genomes and the 99% and 95% observed frequency core genomes of 0.73 and 0.77 respectively, and 0.61 between the 99% and 95% core genomes (Figure S4c). Altogether, these analyses suggest that this estimation approach yields 1) genome-specific gene recovery rates correlated with common assembly quality metrics, 2) estimated frequencies that are robust to sampling and consistent with the existence of core genomes, 3) frequency distributions with a more consistent shape than observed distributions, and 4) core genomes of similar size but different content to those defined using traditional approaches. Original values from these analyses are available in Additional file 4.

Assessing the absence of universal core orthogroups

The absence of any universal orthogroups (OGs) present in all core genomes was not sensitive to the 99.99% frequency threshold for core genes. At more relaxed 99.9% and 99% thresholds, all OGs were still missing in at least 7 and 5 core genomes, respectively (Figure S7a-b). Furthermore, 128 core genomes were missing at least one of the 28 highly conserved OGs (those found in >90% of core genomes) and were from a wide range of species not limited to a specific phylogenetic group (Figure S7c). An alternate approach for identifying universal core OGs by looking at each OG's minimum estimated frequency had similar results: only five OGs had frequency > 90% in all species (maximum 94%) and 20 OGs had frequency > 50% in all species, which were also predominantly ribosomal or other translation-associated proteins (Figure S7d).

Comparison between LBCA core genomes reconstructed under asymmetric Wagner parsimony with Count vs. GLOOME

LBCA core genomes were reconstructed with GLOOME [25] using the same input data used for parsimony-based ancestral reconstruction with Count.jar [23]: core orthogroup (OG) presence/absence calls per species and the species phylogenetic tree. The GLOOME parameter file was generated by providing the input files to the GLOOME server (https://gloome.tau.ac.il/) under default settings (fixed gain/loss ratio, Gamma-distributed rates of variation), and ancestral states were computed locally using GLOOME version "gainLoss.VR01.266". Finally, GLOOME-based LBCA core genomes were computed for different minimum posterior probabilities (MPPs) to accept an OG into the LBCA core genome: 0.5, 0.9, 0.99, 0.999, and 0.9999.

Parsimony-based and GLOOME-based LBCA core genomes were similar in a manner consistent with their respective thresholds for accepting OGs as present in ancestral species (minimum gain/loss ratio or "g" for parsimony, MPP for GLOOME). While the maximal GLOOME LBCA core genome was relatively large (1,783 OGs at MPP = 0.5 vs. 1,301 OGs at g = 2.0

based on parsimony, Figure S9a), applying more stringent probability thresholds to GLOOME yielded LBCA core genomes similar to those based on parsimony. For example, at MPP = 0.9999, the GLOOME LBCA core genome had a maximum Jaccard similarity of 0.72 with the parsimony-based LBCA at g = 0.95, while the GLOOME LBCA with a less stringent threshold of MPP = 0.99 had a maximum Jaccard similarity of 0.77 with a similarly less stringent parsimony-based LBCA at g = 1.9 (Figure S9b). Regarding individual genes, the posterior probability from GLOOME of an OG being in the LBCA core genome was strongly correlated with the minimum gain/loss ratio at which the OG would be in the LBCA core genome under the parsimony approach (Spearman correlation -0.773, Figure S9c). Furthermore, LBCA core OGs recovered at $g \leq 1$ had minimum and mean GLOOME posterior probabilities of 0.9921 and 0.9997, respectively, confirming their label as "high-confidence" LBCA core OGs. Overall, the two methods yield similar LBCA core genomes with variations limited to the lower confidence LBCA core OGs (i.e. those with smaller posterior probabilities from GLOOME or only recovered with larger gain/loss ratios for the parsimony-based approach). GLOOME posterior probabilities are available in Additional file 5.

Ambiguity in mapping between COG and KEGG orthogroups

While most metabolic COG orthogroups mapped to exactly one or zero KEGG orthogroups, there were two instances where a COG orthogroup could not be unambiguously assigned to KEGG orthogroup to determine the presence of a reaction in the LBCA. For phosphogluconolactonase *pgl* in the pentose phosphate pathway (Figure 5b), the COG orthogroup COG0363 represents both *pgl* and *nagB*, glucosamine-6-phosphate deaminase, making it impossible to determine unambiguously whether *pgl* is present in the LBCA core genome with this approach. Similarly, in methionine biosynthesis, the COG orthogroup COG0626 represents both *metB* and *metC*, making it impossible to distinguish the two steps' presence in the LBCA core genome using the current approach. In the latter case, regardless of whether *metB* and/or *metC* are active, methionine could also be produced through an acetyl-homoserine intermediate via *metX* and *metY*, instead of through succinyl-homoserine.

Supplemental Figures

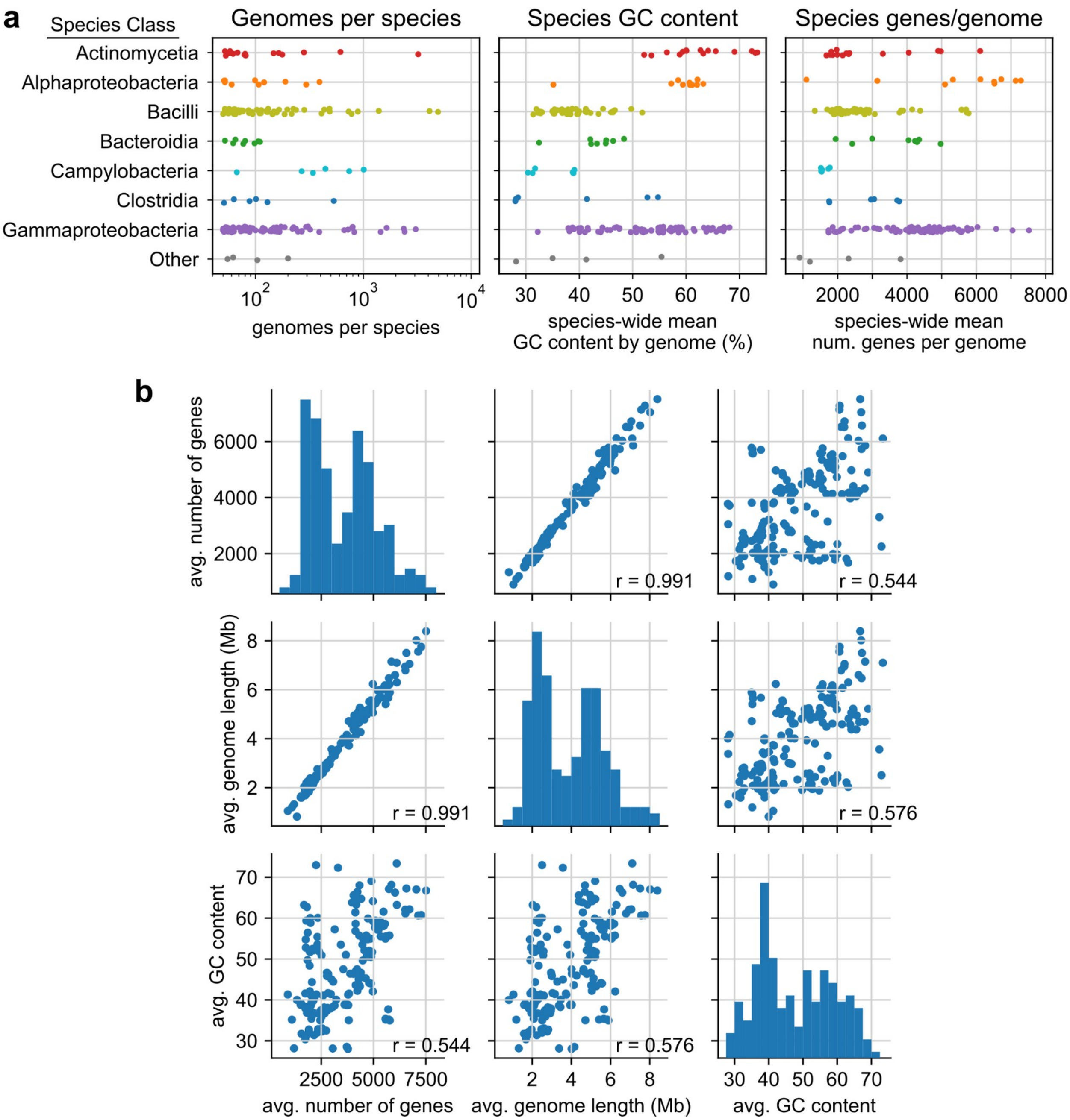

Figure S1: Properties of selected genomes by species and phylogenetic class. a) Genome collection sizes, mean GC content, and mean genes per genome for each species by phylogenetic class. b) Distribution of species-wide averages in number of genes per genome, genome length, and GC content. Diagonal panels show histograms for individual variables, and off-diagonal panels show scatterplots between each pair of variables. Pearson correlation coefficients are shown for each pair.

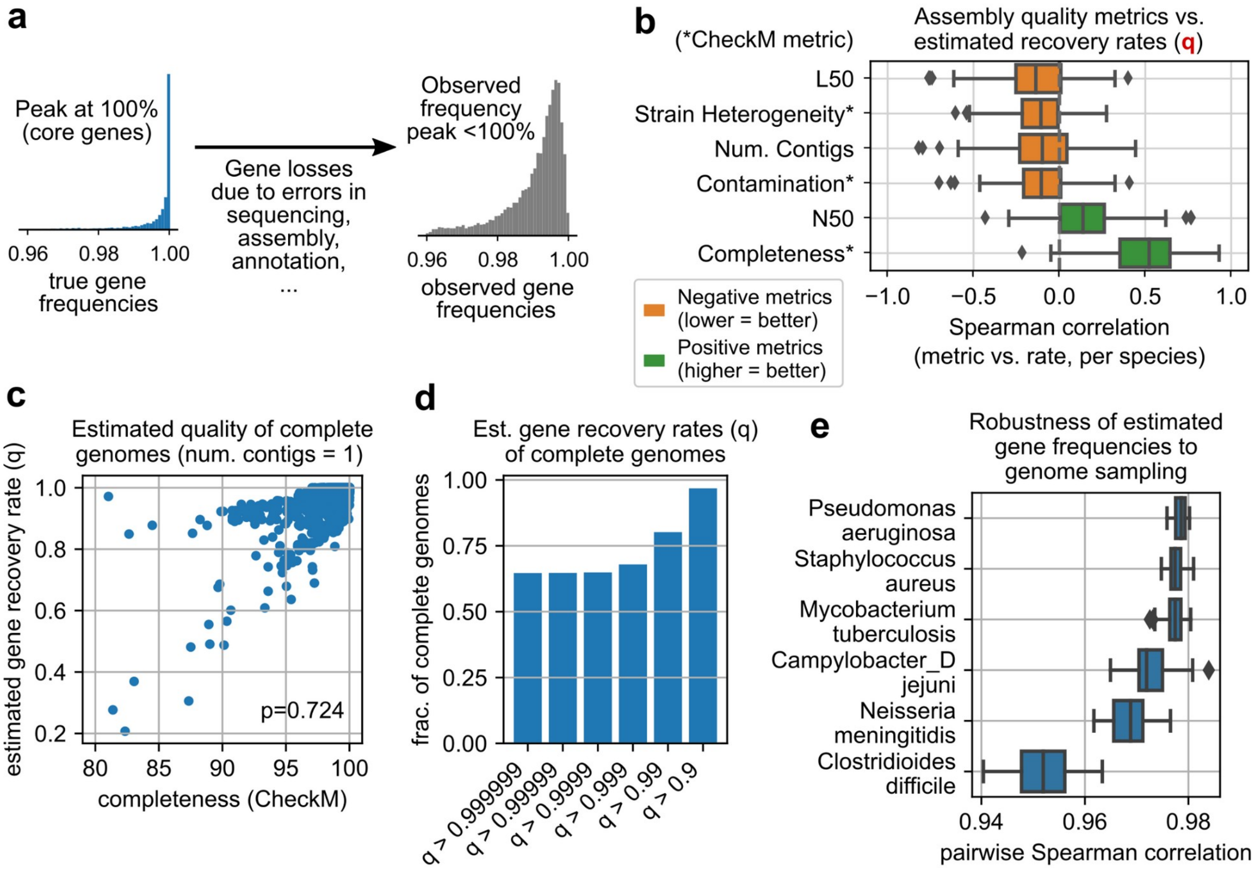

Figure S2: Analysis of estimated gene recovery rates and gene frequencies with respect to genome quality and robustness to random genome sampling. a) Gene losses due to artifacts in the genome assembly process shift the distribution of observed gene frequencies such that the most common frequency for potential core genes is not 100%. b) Distribution of correlations between estimated recovery rates and genome assembly quality metrics across 183 species' pangenomes. c) Comparison of CheckM completeness and estimated gene recovery rates for complete genomes (num. contigs = 1). d) Fraction of complete genomes above various gene recovery rate thresholds. e) Robustness of estimated frequencies to genome sampling for selected species. Pairwise Spearman correlations between estimated gene frequencies calculated from randomly sampled genome sets (90% of genomes were sampled 10 times, per species). Species are sorted by median Spearman correlation.

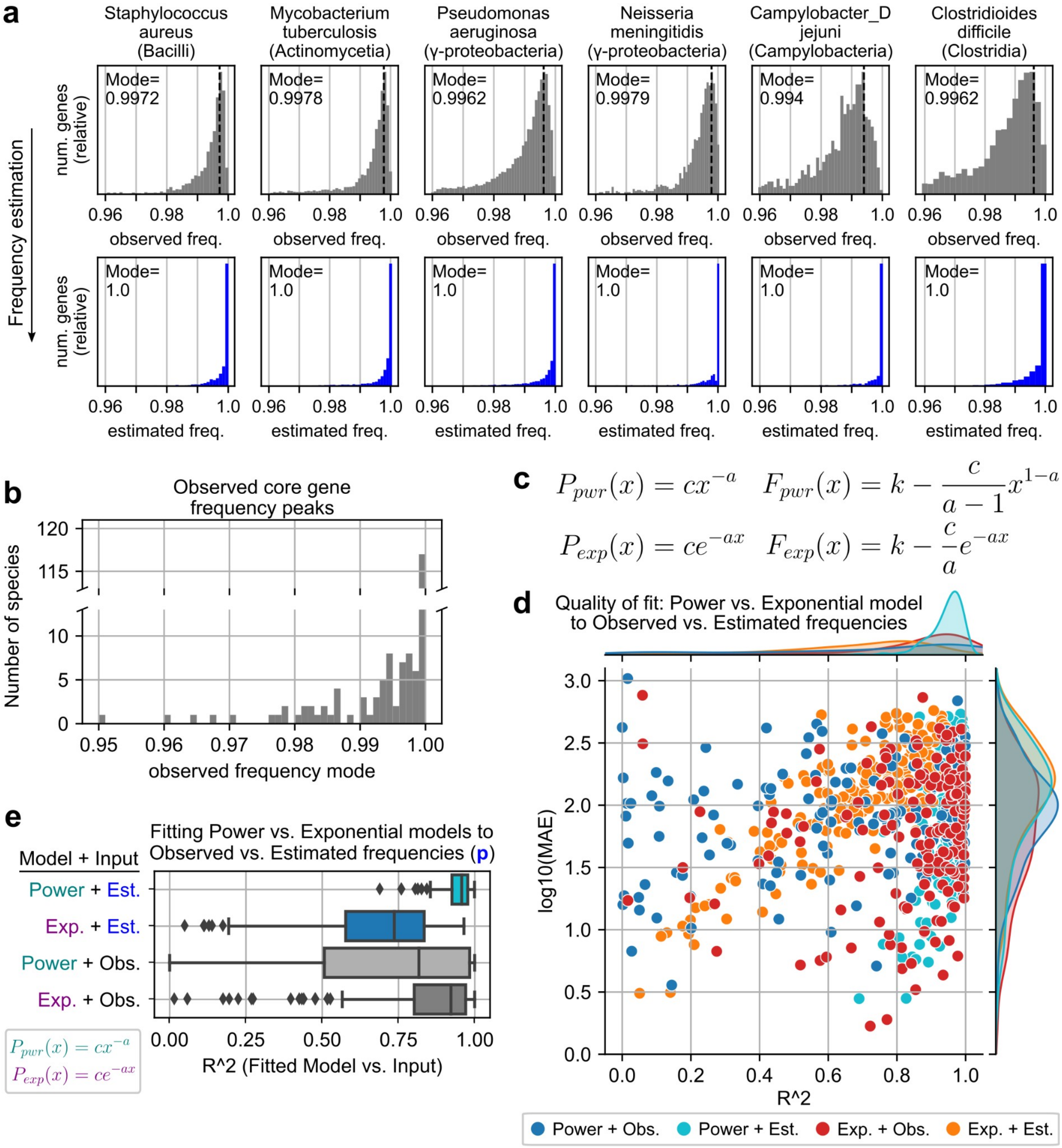

Figure S3: Analysis of estimated gene frequency distributions. a) Comparison of observed and estimated gene frequencies near 100% for selected species, sorted by number of genomes and labeled with phylogenetic class. Modes were calculated using bins of size 1/number of genomes. b) Distribution of observed frequency modes for all 183 species across genes with observed frequency > 50%. c) Power and exponential functions for modeling frequency distributions P(x) and corresponding cumulative distributions F(x), where c, a, and k are fitted parameters. d) Quality of fit (R^2 and log mean absolute error) between either observed or estimated frequencies and either the power or exponential model. e) Distribution of R^2 between observed or estimated frequencies and either the power or exponential model.

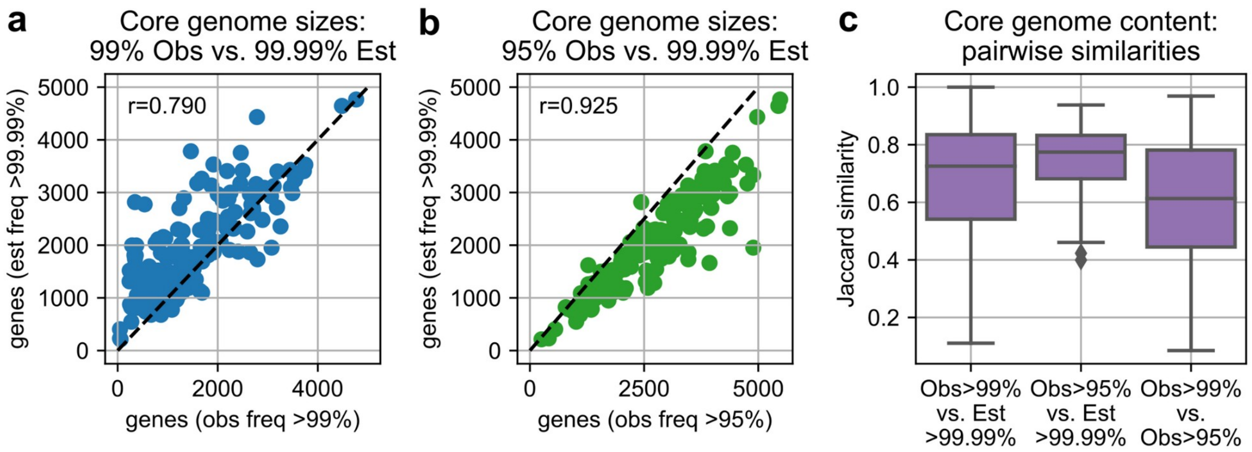

Figure S4: Comparisons between three definitions of core genome across 183 species. a) Pearson correlation between the number of genes with observed frequency >99% vs. estimated frequency >99.99%. b) Pearson correlation between the number of genes with observed frequency >95% vs. estimated frequency >99.99%. c) Pairwise Jaccard similarities between the set of genes with either estimated frequency >99.99%, observed frequency >99%, or observed frequency > 95%.

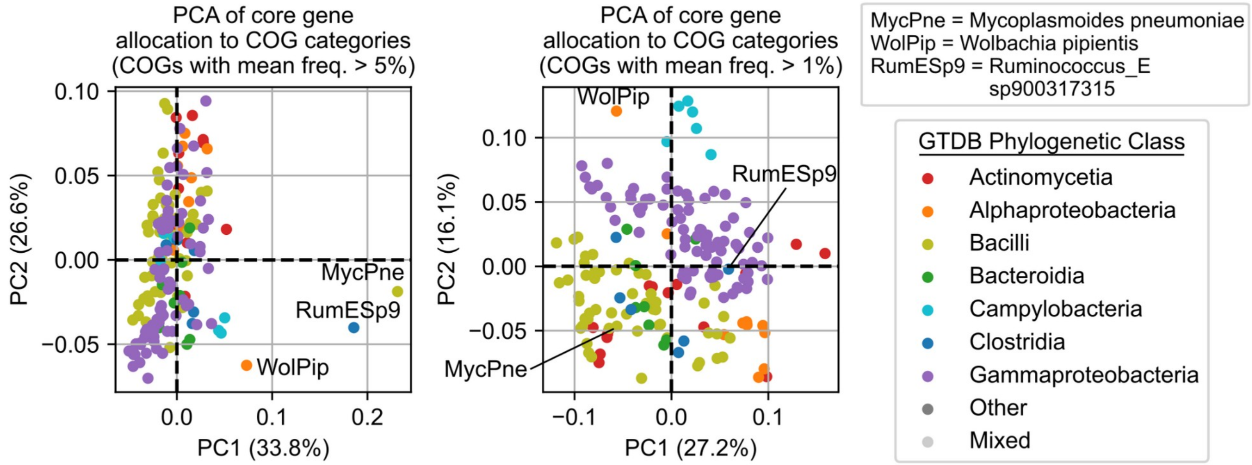

Figure S5: Principal component analysis of core genome allocation of genes to functional categories. PCA was applied to a matrix where each species was represented as a row vector and each element corresponds to the fraction of the species' core genes that was assigned to a specific COG functional category. On the left, COG categories with frequency < 5% were grouped together and on the right COG categories with frequency < 1% before applying PCA, and projections onto the first two components and percent of variance explained are shown. Outliers from the left plot are labeled in both plots.

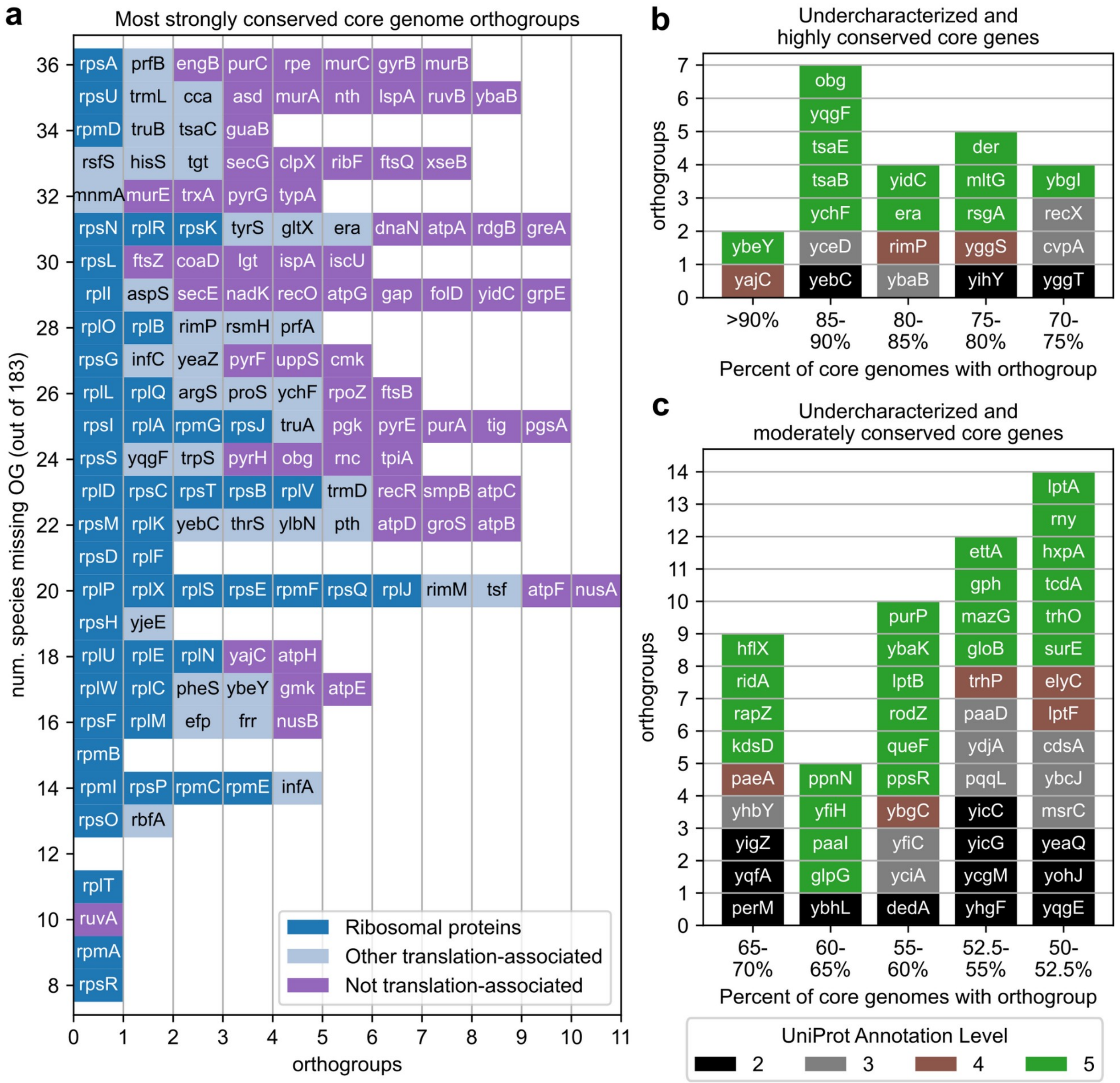

Figure S6: Survey of orthogroups frequently observed in core genomes. a) Orthogroups observed in at least 80% of core genomes, sorted by frequency of observation and function. No orthogroup was observed in more than 175 core genomes (i.e. missing in fewer than 8). b-c) Under-characterized orthogroups observed in at least 50% of core genomes. Orthogroups were identified as potentially under-characterized if assigned to COG category “S: Function unknown” or associated with a y-gene. Orthogroup gene name and level of annotation was corroborated with UniprotKB.

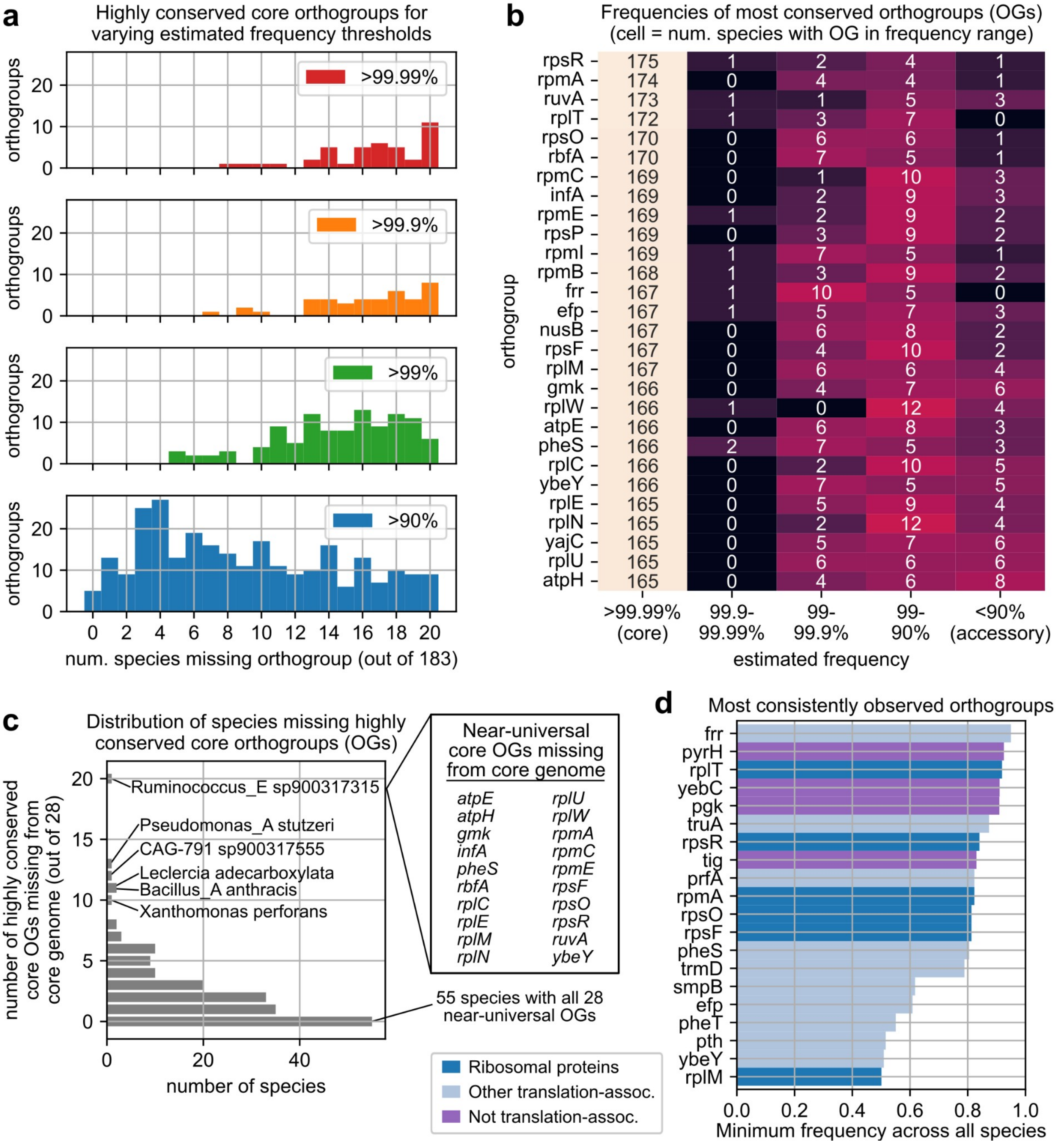

Figure S7: Sensitivity of conserved orthogroups to core genome frequency threshold. a) Distribution of orthogroups commonly observed in core genomes for different frequency thresholds for defining core genes. b) Distribution of estimated frequencies of the most conserved orthogroups. c) Distribution of the number of missing, highly conserved core orthogroups across species. Species missing the most such orthogroups are labeled, and the 20 highly conserved orthogroups missing from *Ruminococcus_E sp900317315* are shown. d) Orthogroups with the highest minimum estimated frequency across all 183 species.

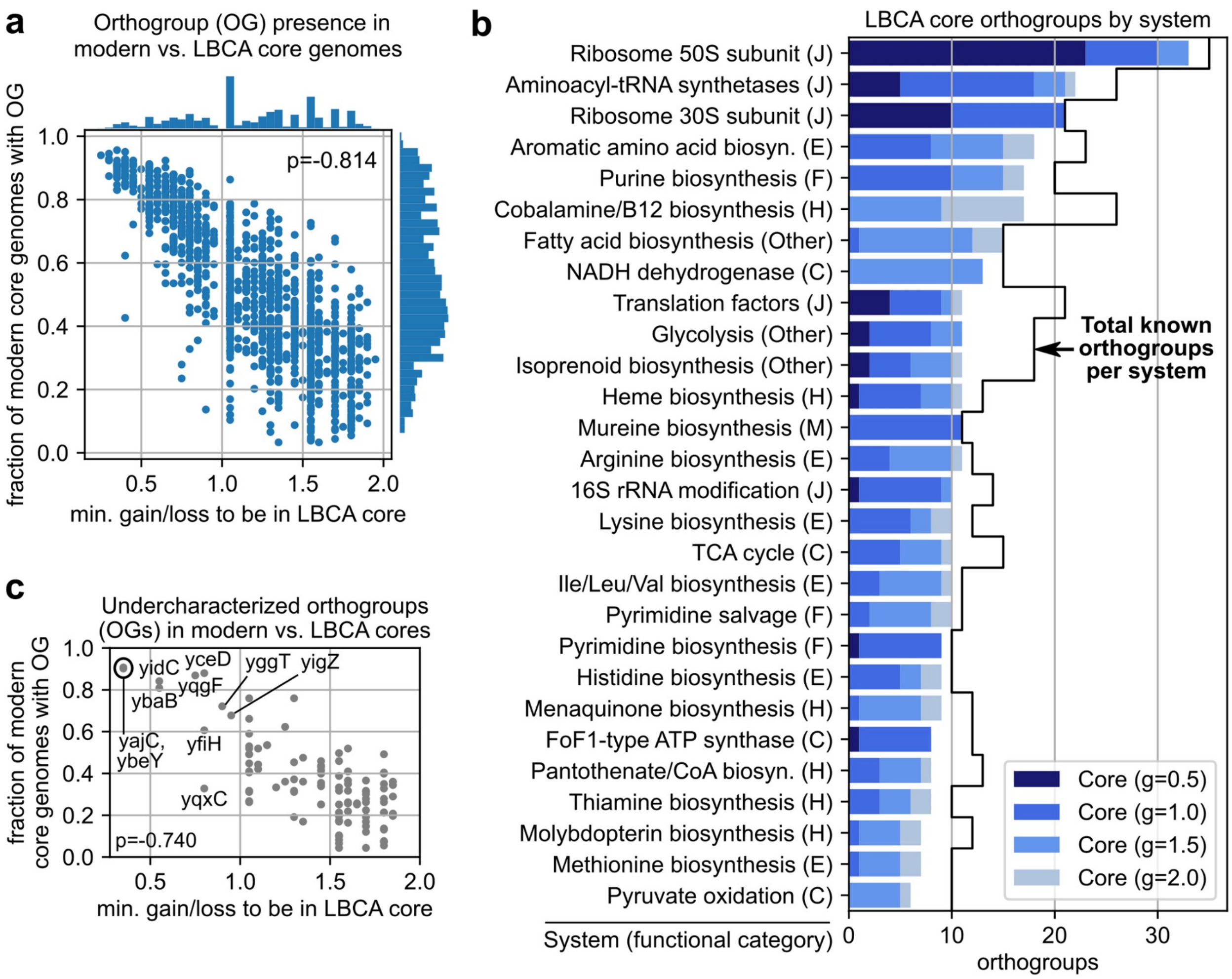

Figure S8: Additional analyses of the LBCA core genome gene content. a) Relationship between the minimum gain/loss penalty during ancestor reconstruction (g) for an orthogroup to be predicted to be in the LBCA core genome vs. the frequency at which it is observed in modern core genomes. The Pearson correlation coefficient is shown. b) Expanded distribution of LBCA core orthogroups by biological system and minimum gain/loss penalty to be observed. Systems with at least 10 orthogroups of which at least 50% are present in the LBCA core genome at g = 2.0 are shown. c) Under-characterized LBCA core orthogroups, by minimum gain/loss penalty and fraction of 183 modern bacterial core genomes with the orthogroup. Orthogroups observed for g < 1.0 are labeled, and the Pearson correlation coefficient is shown.

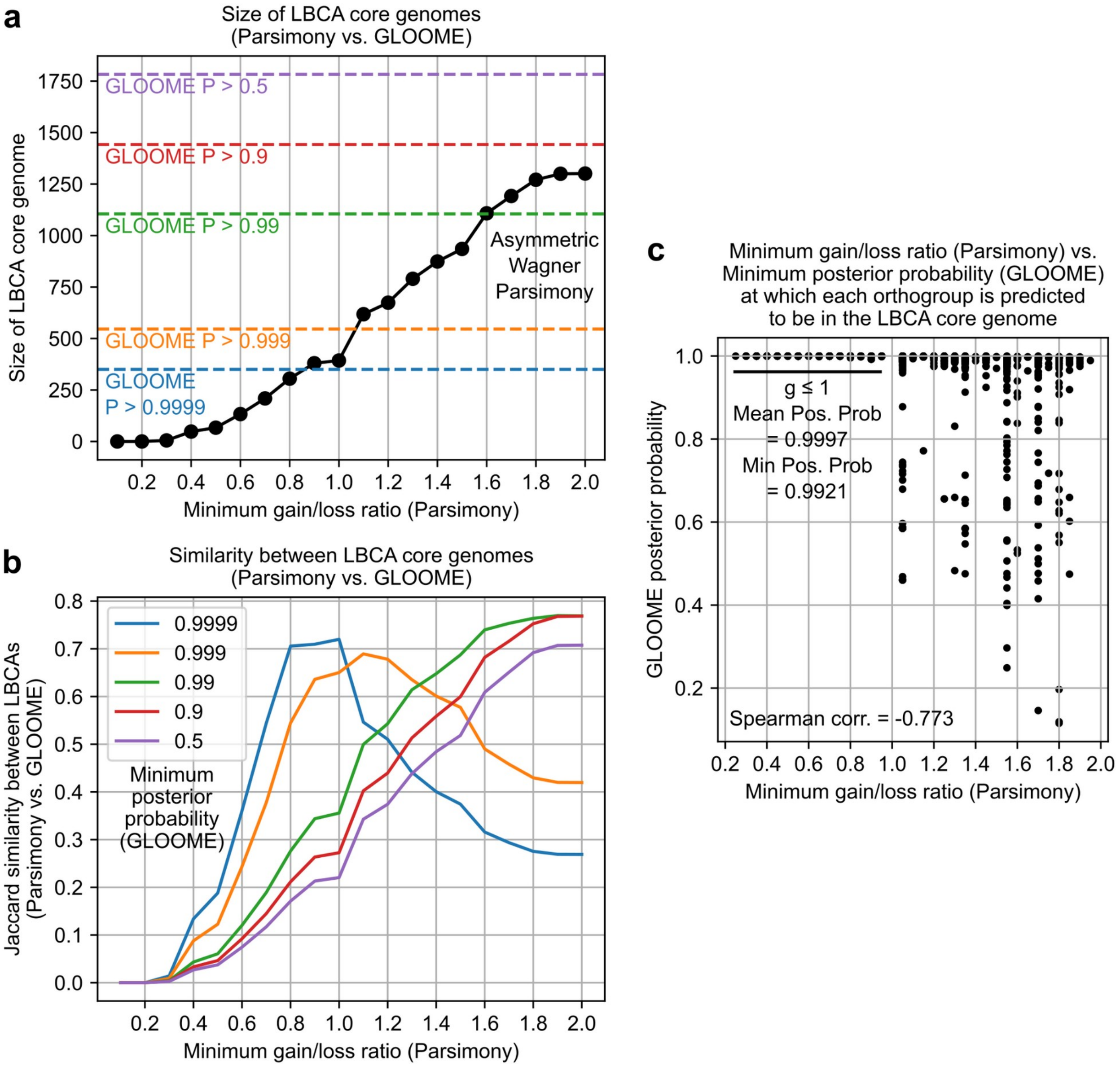

Figure S9: Comparison between LBCA core genomes reconstructed under asymmetric Wagner parsimony with Count vs. maximum likelihood with GLOOME. a) Reconstructed LBCA core genome sizes by method and threshold. Stringency in calling an orthogroup (OG) as an LBCA core OG is controlled by the minimum gain/loss ratio (for parsimony models, solid black line) or minimum posterior probability "P" (for GLOOME, dashed lines). b) Jaccard similarity between LBCA core genomes by method and threshold. Each curve represents the similarity between the GLOOME LBCA core genome for a fixed P vs. parsimony-based LBCA core genomes of increasing minimum gain/loss ratio. c) Correlation between the GLOOME- and parsimony-based thresholds (minimum posterior probability, minimum gain/loss ratio) for calling each orthogroup a LBCA core OG.

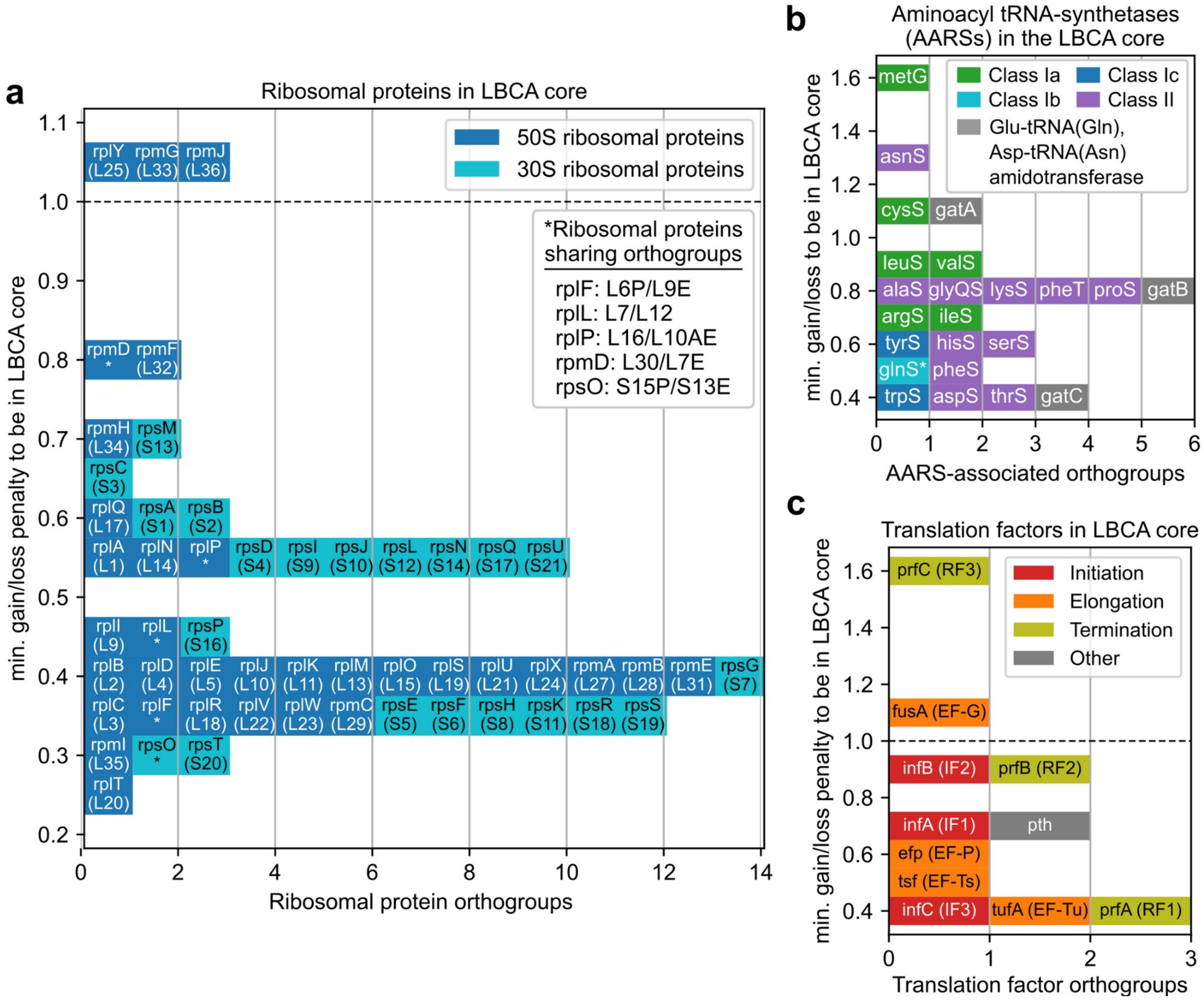

Figure S10: Distribution of LBCA core orthogroups related to ribosomal proteins, aminoacyl-tRNA synthetases, and translation factors. The minimum gain/loss penalty (during ancestor reconstruction via asymmetric Wagner parsimony) at which each orthogroup is predicted to be in the LBCA core genome is shown for a) ribosomal proteins, b) aminoacyl-tRNA synthetases, and c) translation factors. Predicted gene names and corresponding protein names are shown. Starred gene glnS* refers to a single orthogroup containing both GluRS and GlnRS.

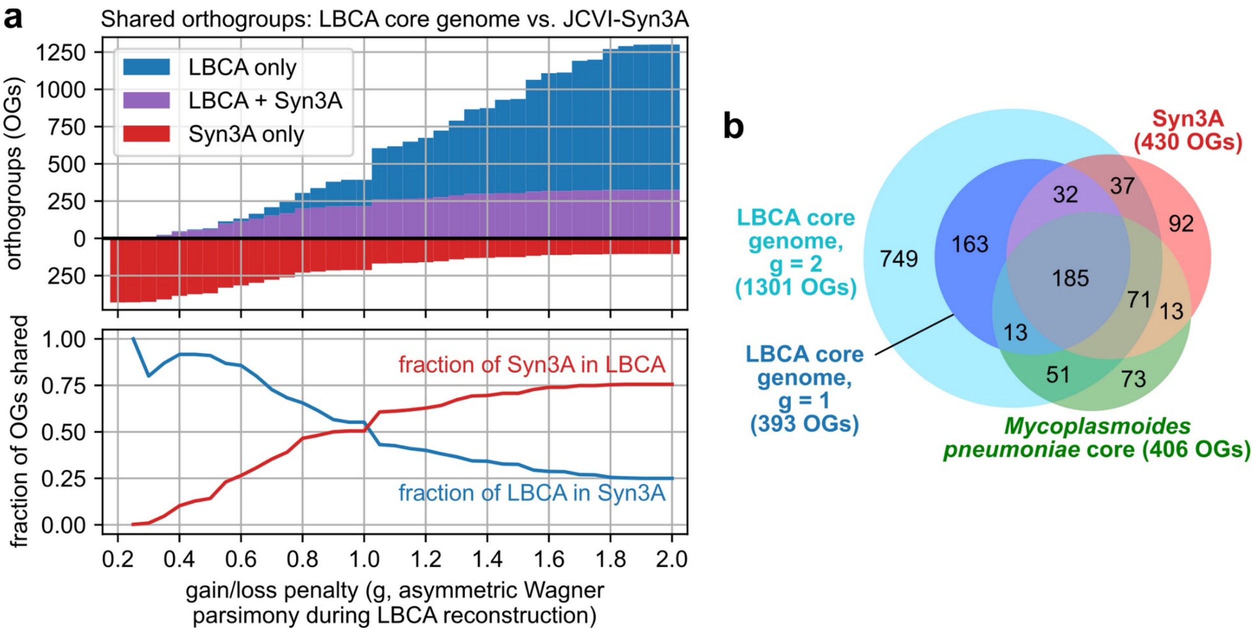

Figure S11: Breakdown of gene overlap between the LBCA core genome and genome of minimal organism JCVI-Syn3A. a) Shared orthogroups (OGs) between the LBCA core genome and Syn3A as the gain/loss penalty ratio (g) is increased during ancestral reconstruction through asymmetric Wagner parsimony (resulting in larger, less stringently defined LBCA core genomes). b) Shared OGs between Syn3A, the LBCA core genome (at g = 1.0 and g = 2.0), and the core genome of *Mycoplasmoides pneumoniae*.

Supplemental Tables

Table S1: 28 under-characterized orthogroups frequently observed in core genomes. Columns left-to-right correspond to predicted gene name, fraction of core genomes with the orthogroup, COG ID, Uniprot ID for *E. coli* member when available, COG functional category, Uniprot annotation level as of Sept. 2022, and orthogroup annotation.

Gene Name	Frac. Core	Orthogroup	Uniprot ID	COG category	Annot. Level	Annotation
yebC	0.8798	COG0217	P0A8A0	K	2	Transcriptional and/or translational regulatory protein YebC/TACO1
yceD	0.8798	COG1399	P0AB28	S	3	23S rRNA accumulation protein YceD (essential in plants, uncharacterized in bacteria)
ybaB	0.8087	COG0718	P0A8B5	S	3	DNA-binding nucleoid-associated protein YbaB/EfbC
yihY	0.7596	COG1295	P0A8K8	S	2	Uncharacterized membrane protein, BrkB/YihY/UPF0761 family (not an RNase)
cvpA	0.7377	COG1286	P08550	S	3	Colicin V production accessory protein CvpA, regulator of purF expression and biofilm formation
yggT	0.7213	COG0762	P64564	S	2	Cytochrome b6 maturation protein CCB3/Ycf19 and related maturases, YggT family
recX	0.7104	COG2137	P33596	S	3	SOS response regulatory protein OraA/RecX, interacts with RecA
yhbY	0.6940	COG1534	P0AGK4	J	3	RNA-binding protein YhbY
yigZ	0.6776	COG1739	P27862	S	2	Putative translation regulator, IMPACT (imprinted ancient) protein family
perM	0.6667	COG0628	P0AFI9	S	2	Predicted PurR-regulated permease PerM
yqfA	0.6612	COG1272	P67153	S	2	Predicted membrane channel-forming protein YqfA, hemolysin III family
ybhL	0.6230	COG0670	P0AAC4	S	2	Integral membrane protein YbhL, putative Ca2+ regulator, Bax inhibitor (BI-1)/TMBIM family
yciA	0.5902	COG1607	P0A8Z0	I	3	Acyl-CoA hydrolase
dedA	0.5847	COG0586	P0ABP6	S	2	Membrane integrity protein DedA, putative transporter, DedA/Tvp38 family
yfiC	0.5574	COG4123	P31825	S	3	tRNA1(Val) A37 N6-methylase TrmN6
pqqL	0.5410	COG0612	P31828	S	3	Predicted Zn-dependent peptidase, M16 family
yhgF	0.5355	COG2183	P46837	K	2	Transcriptional accessory protein Tex/SPT6
ycgM	0.5355	COG0179	P76004	Q	2	2-keto-4-pentenoate hydratase/2-oxohepta- 3-ene-1,7-dioic acid hydratase (catechol pathway)
yicG	0.5355	COG2860	P0AGM2	S	2	Uncharacterized membrane protein
yicC	0.5301	COG1561	P23839	S	2	Uncharacterized stationary-phase protein YicC, UPF0701 family
ydjA	0.5301	COG0778	P0ACY1	C	3	Nitroreductase
paaD	0.5301	COG2151	P76080	S	3	Metal-sulfur cluster biosynthetic enzyme

yohJ	0.5246	COG1380	P60632	S	2	Putative effector of murein hydrolase LrgA, UPF0299 family
yeaQ	0.5191	COG2261	P64485	S	2	Uncharacterized membrane protein YeaQ/YmgE, transglycosylase-associated protein family
msrC	0.5191	COG1956	P76270	T	3	GAF domain-containing protein, putative methionine-R-sulfoxide reductase
cdsA	0.5191	COG4589	O31752*	S	3	Predicted CDP-diglyceride synthetase/phosphatidate cytidylyltransferase
yqgE	0.5137	COG1678	P0A8W5	K	2	Putative transcriptional regulator, AlgH/UPF0301 family
ybcJ	0.5027	COG2501	P0AAS7	S	3	Ribosome-associated protein YbcJ, S4-like RNA-binding protein

*Best match was to *B. subiltis* gene shown here, nearest *E. coli* gene is *ynbB* with Uniprot ID P76091.

Table S2: Translation-associated orthogroups from three systems only present in the LBCA core genome when reconstructed with gain/loss penalties (g) above 1.0.

orthogroup	g	system	gene	comments
COG1825	1.05	Ribosome 50S subunit	*rplY* (L25)	Missing across several bacterial phylogenetic classes [22]
COG0267	1.05	Ribosome 50S subunit	*rpmG* (L33)	Missing in several bacterial species, compensatory paralogs such as *rpmGB* have been observed [22]
COG0257	1.05	Ribosome 50S subunit	*rpmJ* (L36)	Missing in several bacterial species, compensatory paralogs such as *ykgO* have been observed [22]
COG1358	>2.00	Ribosome 50S subunit	*rpl7Ae* (L7ae)	Essential protein in archaea only, homologs observed in only a limited set of bacteria [37]
COG0215	1.05	Aminoacyl-tRNA synthetases	*cysS* (CysRS)	Missing in some methanogenic archaea, Cys-tRNA generated through a o-phosphoseryl-tRNA intermediate via *SepRS* [38]
COG0017	1.25	Aminoacyl-tRNA synthetases	*asnS* (AsnRS)	Missing in several bacteria and archaea, Asn-tRNA generated by modifying misacylated Asp-tRNA(Asn) via *GatABC* [39]
COG0143	1.55	Aminoacyl-tRNA synthetases	*metG* (MetRS)	Complex evolutionary history, may result in incomplete coverage by a single orthogroup [27]
COG0480	1.05	Translation factors	*fusA* (EF-G)	EF-G subfamilies are highly diverse, may result in incomplete coverage by a single orthogroup [28]
COG4108	1.60	Translation factors	*prfC* (RF-3)	Predicted to have emerged post-LBCA as an offshoot of EF-G [41]
