# Supplementary material for: Reconstruction of the last bacterial common ancestor from 183 pangenomes reveals a versatile ancient core genome
Source: Genome Biol. 2023 Aug 8;24:183. doi: 10.1186/s13059-023-03028-2 (PMC10411014; doi:10.1186/s13059-023-03028-2)
Supplement: Supplementary file 7 — Additional file 7. Review history. [file 13059_2023_3028_MOESM7_ESM.docx]

Review History

**First round of review**

**Reviewer 1**

**Are you able to assess all statistics in the manuscript, including the appropriateness of statistical tests used?** No, I do not feel adequately qualified to assess the statistics.

**Comments to author:**

Hyun and Palsson develop and apply a pangenomic approach to reconstruct ancestral bacterial genomes. After reconstructing the pangenomes of 183 diverse bacterial species, they superimpose these pangenomes on the bacterial phylogenetic tree and reconstruct the pangenome of the LBCA, coming to the conclusion that the ancestral bacterial pangenome was (nearly) as complex as the extant pangenomes and encompassed most of the signature bacterial functionalities.

The work is carefully done, and although the results are not at all unexpected, this by far the most comprehensive reconstruction of this type, and for that reason, I find the article important.

I do have a more or less major methodological comment, though. For the reconstruction of the LBCA pangenome, the authors only apply asymmetric Wagner parsimony as implemented in COUNT.

I strongly suggest supplementing this method by maximum likelihood reconstruction using GLOOME https://pubmed.ncbi.nlm.nih.gov/20876605/. At least in the experience of this reviewer,

this is a superior method even if the differences are often minor. Comparing the results obtained with two independent methods would make the conclusions more robust and compelling.

Other than that. I only have minor comments, with the caveat that my qualifications do not allow me to responsibly assess the validity of the statistical procedure the authors developed to maximize the likelihood of pangenome reconstructions.

Nevertheless, the logic of the procedure is straightforward, and I expect it work well.

-I believe it would benefit the reader if the first subsection of the Result explained how the 183 bacterial species included in the reconstruction were selected.

-I would not use the term 'theory' with regard to various views of ancestral genomes - these really are hypotheses or scenarios.

**Reviewer 2**

**Are you able to assess all statistics in the manuscript, including the appropriateness of statistical tests used?** Yes, and I have assessed the statistics in my report.

**Comments to author:**

Hyun and Palsson present a very interesting investigation into the gene content of the last bacterial common ancestor LBCA, using 183 pangenomes from across the bacteria tree. The paper is of broad general interest and is a substantial advance beyond previous work. They find that "The LBCA core genome is distinct from any individual modern core genome but has many fundamental biological systems intact, especially those involving translation machinery and biosynthetic pathways to all major nucleotides and amino acids." This is an interesting and important finding that tells us something we already suspect intuitively (LBCA had to be able to make amino acids and nucleic acids) which means that their method is very probably doing something right. The paper is attractively presented and is an excellent contribution to the literature, although the paper could be substantially strengthened just by adding a few statements or passages in the text.

1. I would like to start with some effervescing praise of this paper's good scientific practice. What I like most about this paper is the use of a simple model (Wagner parsimony with different gain loss ratios) to address the question and honest up-front presentation of what the model and the key parameter do with respect to our estimate of LBCA genome content. A very small increase in the gain to loss ratio at about 1.0 (Figure 3a) generates a 50% increase in LBCA genome size. They show this effect and they talk about it. The authors are to be congratulated and commended for showing this effect. Why am I so happy about this seemingly trivial point? Because it has become commonplace in newspaper journals for teams who are inferring ancestral gene presence or absence to obfuscate in the text and hide in the results the nuts and bolts of what their more complicated methods are doing. The prime example of that "hiding the effects of a complicated method" is Coleman et al. (A rooted phylogeny…Science 2021) where a massively overparameterized maximum likelihood method was used to infer the position of the root in the bacterial tree. The Coleman results hinged crucially upon exactly the same parameter as in this paper: the gain/loss ratio (they called it the duplication to transfer ratio but it boils down to much the same thing). But Coleman et al. (2021) hid their effects in a tome of supplemental. Bremer et al. (Realistic gene transfer ratios… https://doi.org/10.3390/life12070995) had to show everyone what the problem was, because Coleman et al. were hiding it. What I am trying to say here is that Hyun and Palsson set an outstanding example with this paper by really digging into — and reporting — the nuts and bolts of what their method is doing with respect to the ancestral genome results that it returns. There is no need to discuss Coleman or its problems here. I am just saying that the open dealings with the method here is a very strong argument for publication of this paper with only minor text modifications, in this referee's opinion. Why do I make such a big fuss here? Because other referees might swoop in claiming that likelihood methods need to be used, which is not true. The present method delivers very interesting, useful and reasonable results that can be debated or analyzed with other methods in public after the paper is published, not beforehand.

2. It should be stated somewhere that the sample, constrained by pangenome availability, is somewhat biased towards proteobacteria and aerobes, and that the choice of sample can (obviously) influence the result of any method inferring an ancestral state. There is no need to demonstrate that effect here, others can investigate that once this data is published.

3. The recovery of full amino acid and nucleic acid synthesis is both a good result and a good positive control for the method. Why a positive control? Because unless LBCA was living off of amino acids and nucleic acids synthesized abiotically or synthesized by archaea, it had to have complete amino acid and base biosynthetic pathways. They do explicitly state that ref. 3 recently found a similar result. In this context, Wimmer et al. 2021 identified the set of roughly 400 chemical reactions (KEGG reactions) that are required to synthesize all of the amino acids, bases, and 18 cofactors from H2, CO2, H2S, NH3, water and phosphate (The autotrophic core… 10.3390/microorganisms9020458). Those *reactions* had to be present in LUCA because LUCA had 20 amino acids and RNA. What Wimmer et al. did not identify were the *genes* or the *enzymes* for those reactions, so a major contribution of the present work is to provide a list of genes and enzymes that trace to LBCA's core biosynthetic metabolism using robust methods. This is interesting because LUCA might well have used compounds provided geochemically or other abiotic synthesis (life started out from nothing but CO2, so there did exist a process of enzyme accumulation), and in some theories LBCA might have done so as well. What Hyun and Palsson show is a genome based result that LBCA was a fully fledged prototroph with regard to amino acids and bases. That is a major insight, actually, that has not emerged from any other gene or genome based study to my knowledge. One might mention it. This is probably why LBCA can do more than SYN-3A, which lives with the help of compounds made by other cells. [Cofactors might be more complicated than amino acids and bases, because so many organisms in the sample have cofactor auxotrophies. Getting into cofactors is something for a different paper.]

4. Energy metabolism does not really reconstruct to LBCA from my read of this paper, which is not surprising because of the prevalence of aerobes in the present sample and because of the simply staggering diversity of energy conserving protein complexes in bacteria (see for example papers by Manuera M. Pereira: Marreiros et al Biochimica et Biophysica Acta 1857 (2016) 1039-1067; Calisto and Pereira, Biochemical Society Transactions (2021) 49 2669-2685 https://doi.org/10.1042/BST20210462). With Wagner parsimony (or any other method, actually, the most frequent and widely distributed proteins will tend to reconstruct to LBCA, such that with highly diverse functions like energy metabolism and oxygen effects, energy metabolism becomes difficult to trace to LBCA.

5. It might be helpful for some readers to recapitulate in the discussion that the gain loss ratio generates a series of LBCA reconstructions of differing size and completeness with respect to core biosynthetic functions. The picture is however consistent and biologically very reasonable.

Other than those thoughts, this referee's recommendation is publish with very minor modifications.

**Authors Response**

**Point-by-point responses to the reviewers’ comments:**

**Reviewer #1**

Hyun and Palsson develop and apply a pangenomic approach to reconstruct ancestral bacterial genomes. After reconstructing the pangenomes of 183 diverse bacterial species, they superimpose these pangenomes on the bacterial phylogenetic tree and reconstruct the pangenome of the LBCA, coming to the conclusion that the ancestral bacterial pangenome was (nearly) as complex as the extant pangenomes and encompassed most of the signature bacterial functionalities.

The work is carefully done, and although the results are not at all unexpected, this by far the most comprehensive reconstruction of this type, and for that reason, I find the article important. I do have a more or less major methodological comment, though. For the reconstruction of the LBCA pangenome, the authors only apply asymmetric Wagner parsimony as implemented in COUNT. I strongly suggest supplementing this method by maximum likelihood reconstruction using GLOOME <https://pubmed.ncbi.nlm.nih.gov/20876605/>. At least in the experience of this reviewer, this is a superior method even if the differences are often minor. Comparing the results obtained with two independent methods would make the conclusions more robust and compelling.

Thank you for this valuable suggestion in improving the robustness of our results. We have since added a new analysis comparing our parsimony/COUNT-based LBCA core genomes to those similarly generated using GLOOME.

We find that while GLOOME can be somewhat more permissive than COUNT, the methods readily yield similar LBCA core genomes when adjusting their respective thresholds for controlling LBCA core genome size (minimum gain/loss ratio for COUNT, minimum posterior probability for GLOOME). For example, a strictly-defined GLOOME LBCA (probability > 0.9999) had maximum Jaccard similarity of 0.72 with a COUNT LBCA at a strict g = 0.95, while a more relaxed GLOOME LBCA (probability > 0.99) had maximum Jaccard similarity of 0.77 with a COUNT LBCA at a relaxed g = 1.9. Additionally, the LBCA orthogroups identified by COUNT at the strict g ≤ 1 threshold had minimum and mean posterior probabilities from GLOOME of 0.9921 and 0.9997 respectively, consistent with our work referring to those orthogroups as “high-confidence”. Overall, we believe these results suggest that our COUNT-based LBCA core genomes are generally similar to those from GLOOME, especially among high-confidence orthogroups that were the focus of much of our analysis.

*Results*: Finally, the LBCA core genomes were also compared with those reconstructed using GLOOME, which applies a maximum likelihood approach to reconstruction rather than parsimony-based optimization (Cohen et al. 2010). Under default settings (fixed gain/loss ratio, gamma-distributed rates of variation), LBCA core genomes from GLOOME were consistent with parsimony-based core genomes in terms of both overall gene content and thresholds for individual genes (i.e. minimum gain/loss ratio for parsimony models vs. posterior probability for GLOOME), and OGs recovered at g=1.0 with the parsimony-based approach had mean and minimum GLOOME posterior probabilities of 0.9997 and 0.9921, respectively (Figure S9, see Supplemental Analysis).

*Supplemental Analysis*: LBCA core genomes were reconstructed with GLOOME (Cohen et al. 2010) using the same input data used for parsimony-based ancestral reconstruction with Count.jar (Csurös 2010): core orthogroup (OG) presence/absence calls per species and the species phylogenetic tree. The GLOOME parameter file was generated by providing the input files to the GLOOME server (https://gloome.tau.ac.il/) under default settings (fixed gain/loss ratio, Gamma-distributed rates of variation), and ancestral states were computed locally using GLOOME version “gainLoss.VR01.266”. Finally, GLOOME-based LBCA core genomes were computed for different minimum posterior probabilities (MPPs) to accept an OG into the LBCA core genome: 0.5, 0.9, 0.99, 0.999, and 0.9999.

Parsimony-based and GLOOME-based LBCA core genomes were similar in a manner consistent with their respective thresholds for accepting OGs as present in ancestral species (minimum gain/loss ratio or “g” for parsimony, MPP for GLOOME). While the maximal GLOOME LBCA core genome was relatively large (1,783 OGs at MPP = 0.5 vs. 1,301 OGs at g = 2.0 based on parsimony, Figure S9a), applying more stringent probability thresholds to GLOOME yielded LBCA core genomes similar to those from based on parsimony. For example, at MPP = 0.9999, the GLOOME LBCA core genome had a maximum Jaccard similarity of 0.72 with the parsimony-based LBCA at g = 0.95, while the GLOOME LBCA with a less stringent threshold of MPP = 0.99 had a maximum Jaccard similarity of 0.77 with a similarly less stringent parsimony-based LBCA at g = 1.9 (Figure S9b). Regarding individual genes, the posterior probability from GLOOME of an OG being in the LBCA core genome was strongly correlated with the minimum gain/loss ratio at which the OG would be in the LBCA core genome under the parsimony approach (Spearman correlation -0.773, Figure S9c). Furthermore, LBCA core OGs recovered at g ≤ 1 had minimum and mean GLOOME posterior probabilities of 0.9921 and 0.9997, respectively, confirming their label as “high-confidence” LBCA core OGs. Overall, the two methods yield similar LBCA core genomes with variations limited to the lower confidence LBCA core OGs (i.e. those with smaller posterior probabilities from GLOOME or only recovered with larger gain/loss ratios for the parsimony-based approach). GLOOME posterior probabilities are available in Dataset S4.

**Figure S9: Comparison between LBCA core genomes reconstructed under asymmetric Wagner parsimony with Count vs. maximum likelihood with GLOOME.** a) Reconstructed LBCA core genome sizes by method and threshold. Stringency in calling an orthogroup (OG) as an LBCA core OG is controlled by the minimum gain/loss ratio (for parsimony models, solid black line) or minimum posterior probability “P” (for GLOOME, dashed lines). b) Jaccard similarity between LBCA core genomes by method and threshold. Each curve represents the similarity between the GLOOME LBCA core genome for a fixed P vs. parsimony-based LBCA core genomes of increasing minimum gain/loss ratio. c) Correlation between the GLOOME- and parsimony-based thresholds (minimum posterior probability, minimum gain/loss ratio) for calling each orthogroup a LBCA core OG.

Other than that. I only have minor comments, with the caveat that my qualifications do not allow me to responsibly assess the validity of the statistical procedure the authors developed to maximize the likelihood of pangenome reconstructions. Nevertheless, the logic of the procedure is straightforward, and I expect it work well.

-I believe it would benefit the reader if the first subsection of the Result explained how the 183 bacterial species included in the reconstruction were selected.

Thank you for this recommendation, we have included some of the details from the Methods at the beginning of the Results.

*Results:* Starting from Web of Life (WOL) dataset (Zhu et al. 2019), genomes were filtered based on assembly quality, accounting for number of contigs, total length, CheckM contamination, and CheckM completeness (Parks et al. 2015) (see Methods for details). Filtering yielded 183 bacterial species as defined by GTDB (Parks et al. 2018) with at least 50 high quality genomes each, totaling 54,085 genome assemblies (Dataset S1).

-I would not use the term 'theory' with regard to various views of ancestral genomes - these really are hypotheses or scenarios.

Thank you for this clarification, we have replaced “theories” with “hypotheses” throughout the Introduction and Discussion.

**Reviewer #2**

Hyun and Palsson present a very interesting investigation into the gene content of the last bacterial common ancestor LBCA, using 183 pangenomes from across the bacteria tree. The paper is of broad general interest and is a substantial advance beyond previous work. They find that "The LBCA core genome is distinct from any individual modern core genome but has many fundamental biological systems intact, especially those involving translation machinery and biosynthetic pathways to all major nucleotides and amino acids." This is an interesting and important finding that tells us something we already suspect intuitively (LBCA had to be able to make amino acids and nucleic acids) which means that their method is very probably doing something right. The paper is attractively presented and is an excellent contribution to the literature, although the paper could be substantially strengthened just by adding a few statements or passages in the text.

1. I would like to start with some effervescing praise of this paper's good scientific practice. What I like most about this paper is the use of a simple model (Wagner parsimony with different gain loss ratios) to address the question and honest up-front presentation of what the model and the key parameter do with respect to our estimate of LBCA genome content. A very small increase in the gain to loss ratio at about 1.0 (Figure 3a) generates a 50% increase in LBCA genome size. They show this effect and they talk about it. The authors are to be congratulated and commended for showing this effect. Why am I so happy about this seemingly trivial point? Because it has become commonplace in newspaper journals for teams who are inferring ancestral gene presence or absence to obfuscate in the text and hide in the results the nuts and bolts of what their more complicated methods are doing. The prime example of that "hiding the effects of a complicated method" is Coleman et al. (A rooted phylogeny…Science 2021) where a massively overparameterized maximum likelihood method was used to infer the position of the root in the bacterial tree. The Coleman results hinged crucially upon exactly the same parameter as in this paper: the gain/loss ratio (they called it the duplication to transfer ratio but it boils down to much the same thing). But Coleman et al. (2021) hid their effects in a tome of supplemental. Bremer et al. (Realistic gene transfer ratios… <https://doi.org/10.3390/life12070995>) had to show everyone what the problem was, because Coleman et al. were hiding it. What I am trying to say here is that Hyun and Palsson set an outstanding example with this paper by really digging into — and reporting — the nuts and bolts of what their method is doing with respect to the ancestral genome results that it returns. There is no need to discuss Coleman or its problems here. I am just saying that the open dealings with the method here is a very strong argument for publication of this paper with only minor text modifications, in this referee's opinion. Why do I make such a big fuss here? Because other referees might swoop in claiming that likelihood methods need to be used, which is not true. The present method delivers very interesting, useful and reasonable results that can be debated or analyzed with other methods in public after the paper is published, not beforehand.

We thank the reviewer for their kind remarks and insights into the greater context within which our work belongs. Though we gratefully agree with the reviewer’s comment that our method provides useful standalone results, we have added a brief comparison with a likelihood method (GLOOME) at the recommendation of another reviewer, available in the Supplemental Analysis. We find that our approach and GLOOME are highly consistent for high-confidence LBCA orthogroups that were the focus of our analysis (i.e. those recovered at small gain/loss ratios under parsimony, or those with the highest posterior probabilities from GLOOME). We hope that this new analysis adds to the robustness of our existing results, while variations among lower-confidence LBCA orthogroups may invite more detailed analyses with different methods at this scale.

2. It should be stated somewhere that the sample, constrained by pangenome availability, is somewhat biased towards proteobacteria and aerobes, and that the choice of sample can (obviously) influence the result of any method inferring an ancestral state. There is no need to demonstrate that effect here, others can investigate that once this data is published.

Thank you for pointing out this limitation in the current work. We have updated the Discussion to mention this bias and its implications in the context of future analyses.

*Discussion*: We also note that due to the distribution of species for which sufficient genomes were available for pangenome construction, this analysis likely overrepresents aerobes and proteobacteria which can bias the reconstructed LBCA regardless of computational technique. As more genome sequences are generated for a greater diversity of species, future analyses may improve upon both the coverage and scope of this work to reconstruct more distant ancestral organisms with greater confidence.

3. The recovery of full amino acid and nucleic acid synthesis is both a good result and a good positive control for the method. Why a positive control? Because unless LBCA was living off of amino acids and nucleic acids synthesized abiotically or synthesized by archaea, it had to have complete amino acid and base biosynthetic pathways. They do explicitly state that ref. 3 recently found a similar result. In this context, Wimmer et al. 2021 identified the set of roughly 400 chemical reactions (KEGG reactions) that are required to synthesize all of the amino acids, bases, and 18 cofactors from H2, CO2, H2S, NH3, water and phosphate (The autotrophic core… 10.3390/microorganisms9020458). Those *reactions* had to be present in LUCA because LUCA had 20 amino acids and RNA. What Wimmer et al. did not identify were the *genes* or the *enzymes* for those reactions, so a major contribution of the present work is to provide a list of genes and enzymes that trace to LBCA's core biosynthetic metabolism using robust methods. This is interesting because LUCA might well have used compounds provided geochemically or other abiotic synthesis (life started out from nothing but CO2, so there did exist a process of enzyme accumulation), and in some theories LBCA might have done so as well. What Hyun and Palsson show is a genome based result that LBCA was a fully fledged prototroph with regard to amino acids and bases. That is a major insight, actually, that has not emerged from any other gene or genome based study to my knowledge. One might mention it. This is probably why LBCA can do more than SYN-3A, which lives with the help of compounds made by other cells. [Cofactors might be more complicated than amino acids and bases, because so many organisms in the sample have cofactor auxotrophies. Getting into cofactors is something for a different paper.]

Thank you for this detailed reading on the metabolic reconstruction results and providing context to our findings. We have updated the Discussion to mention how our results support the hypothesis of a largely prototrophic LBCA, extending the findings of Wimmer et.al. to reaction-specific orthogroups.

*Results*: As observed in a previous metabolic analysis of the LBCA predicting its ability to synthesize all 4 DNA bases, 4 RNA bases, and 20 amino acids (Xavier et al. 2021), this pangenomic analysis provides further evidence for a prototrophic LBCA through the identification of individual reaction-specific orthogroups that are predicted to be universally conserved within the LBCA species.

4. Energy metabolism does not really reconstruct to LBCA from my read of this paper, which is not surprising because of the prevalence of aerobes in the present sample and because of the simply staggering diversity of energy conserving protein complexes in bacteria (see for example papers by Manuera M. Pereira: Marreiros et al Biochimica et Biophysica Acta 1857 (2016) 1039-1067; Calisto and Pereira, Biochemical Society Transactions (2021) 49 2669-2685 <https://doi.org/10.1042/BST20210462>). With Wagner parsimony (or any other method, actually, the most frequent and widely distributed proteins will tend to reconstruct to LBCA, such that with highly diverse functions like energy metabolism and oxygen effects, energy metabolism becomes difficult to trace to LBCA.

Thank you for this interesting insight. We have added to the Discussion a comment on how parts of energy metabolism (esp. those involving large complexes) are not confidently reconstructed using our approach, and that this may be a consequence of their outsized diversity confounding ancestor reconstruction.

*Discussion*: More broadly, the absence of AKGDH, as well as other energy metabolism complexes (i.e. succinate dehydrogenase, pyruvate dehydrogenase) only being detected for large gain/loss ratios, may be a result of the outsized diversity observed for such modular complexes (Calisto and Pereira 2021) confounding ancestor reconstruction.

5. It might be helpful for some readers to recapitulate in the discussion that the gain loss ratio generates a series of LBCA reconstructions of differing size and completeness with respect to core biosynthetic functions. The picture is however consistent and biologically very reasonable.

Thank you for this recommendation, we have updated our Discussion to explicitly mention that multiple LBCA core genomes were generated, based on different assumptions on the gain/loss ratio.

*Discussion*: We next integrated our core genomes with the Web of Life phylogeny (Zhu et al. 2019) and applied asymmetric Wagner parsimony to generate multiple reconstructions of the LBCA core genome, based on different assumptions regarding the gain/loss ratio or relative tendency towards gene gain vs. loss events along the phylogenetic tree. Even under strict assumptions yielding smaller ancestral core genomes…

Other than those thoughts, this referee's recommendation is publish with very minor modifications.

**Second round of review**

**Reviewer 1**

In the revision, the authors have carefully addressed my comments, improving the manuscript.

I have no further criticisms.
